# Epidemiology, survival, and treatment of acute myeloid and lymphoblastic leukaemia in Germany: a nationwide population-based registry analysis

**DOI:** 10.1016/j.lanepe.2025.101503

**Published:** 2025-10-18

**Authors:** David Baden, Nadine Wolgast, Philipp M. Altrock, Sophie Steinhäuser, Jakob Voran, Thomas Beder, Manuel Hecht, Cornelia Baden, Lorenz Bastian, Cécile Ronckers, Jacqueline Müller-Nordhorn, Soo-Zin Kim-Wanner, Martin Neumann, Lars Fransecky, Gunnar Cario, Christoph Röllig, Alexander Katalinic, Claudia D. Baldus

**Affiliations:** a Department of Medicine II, Haematology and Oncology, University Hospital Schleswig-Holstein, Christian-Albrechts-University, Kiel, Germany; b University Cancer Centre Schleswig-Holstein, University Hospital Schleswig-Holstein, Kiel, Germany; c Clinical Research Unit CATCH ALL (KFO 5010), Kiel, Germany; d Department of Theoretical Biology, Max Planck Institute for Evolutionary Biology, Ploen, Schleswig-Holstein, Germany; e Department of Internal Medicine III, Cardiology and Intensive Care, University Hospital Schleswig-Holstein, Kiel, Germany; f Division of Childhood Cancer Epidemiology, German Childhood Cancer Registry, Institute for Medical Biostatistics, Epidemiology and Informatics, University Medical Centre of the Johannes Gutenberg University, Main, Germany; g Bavarian Cancer Registry, Bavarian Health and Food Safety Authority, Nuremberg, Germany; h Hessian Cancer Registry, Hessian Office for Health and Care, Frankfurt, Germany; i Department of Paediatrics I, ALL-BFM Study Group, Christian-Albrechts University Kiel and University Medical Centre Schleswig-Holstein, Kiel, Germany; j Medical Department I, University Hospital of TU Dresden, Dresden, Germany; k Institute for Social Medicine and Epidemiology, University Hospital Schleswig-Holstein, Christian-Albrechts-University, Lübeck, Germany

**Keywords:** Acute leukaemia, Acute myeloid leukaemia (AML), Acute lymphoblastic leukaemia (ALL), Epidemiology, Outcomes, Socioeconomic differences

## Abstract

**Background:**

Acute leukaemias are rare but highly aggressive malignancies, but only limited population-level data are available for Germany. We aimed to describe epidemiology, survival, and therapies of acute myeloid leukaemia (AML) and acute lymphoblastic leukaemia (ALL) in Germany using nationwide cancer registry data.

**Methods:**

We conducted a population-based analysis of all incident cases of AML and ALL in Germany, identified via ICD codes from mandatory cancer registry reporting, to assess incidence, treatment, and survival outcomes.

**Findings:**

We identified 25,788 patients with AML and 6480 patients with ALL diagnosed between 2016 and 2021 aged 0–101 years. The age-standardized incidence rate was 4.72/100,000 for AML (median age 72.8 years, IQR 61.0–80.3) and 1.36/100,000 for ALL (median age 19.4 years, IQR 5.2–58.6). The three- and five-year overall survival was 29.0% (95% CI: 28.3–29.7) and 23.8% (95% CI: 23.1–24.7) in AML, and 64% (95% CI: 62.2–65.9) and 58% (95% CI: 55.7–60.2) in ALL. Survival was highly dependent on age, with children (0–18 years) showing the highest three-year survival rates in AML (76.4%, 95% CI: 70.2–83.2) and ALL (91.9%, 95% CI: 89.8–94.1) compared to older adults. Moreover, area-based income and social deprivation were linked to survival, with three-year survival reduced by up to 4% in lower-income counties. Based on German federal population estimates, AML cases are expected to rise by 14.6%, while ALL cases will decline by 2.3% between 2020 and 2050.

**Interpretation:**

We provide incidence and survival data to inform future clinical trials, guide resource allocation, and support healthcare planning to improve real-world outcomes and address disparities in acute leukaemia.

**Funding:**

10.13039/501100001659German Research Foundation (DFG).

**Research in context**

**Evidence before this study**

Data on the incidence and survival of acute myeloid leukaemia (AML) and acute lymphoblastic leukaemia (ALL) in Germany and Europe are limited. A PubMed search (up to 31/08/2025) using (“acute myeloid leukaemia” OR “acute lymphoblastic leukaemia”) AND (epidemiology OR “cancer registry” OR “population-based”) AND (incidence OR survival OR mortality) AND (Germany OR Europe) in titles or abstracts retrieved 19 publications, 13 of which report incidence and/or survival of AML or ALL. Over half of these studies were published before 2015 and are based on data collected prior to 2010.

The most comprehensive German estimates of AML and ALL incidence and survival, published in 2013, covered only ≈15% of the population. The EUROCARE-6 study reported 5-year AML survival of 20.6%–26.1% in European countries with similar health spending but focused on adolescents and young adults, lacked treatment details, and used diagnoses from 2006 to 2013. Given improving survival and demographic changes, these data are now likely outdated.

A second search using (“acute myeloid leukaemia” OR “acute lymphoblastic leukaemia”) AND (socioeconomic factors OR deprivation OR inequalities) AND (Germany OR Europe) yielded only three studies, describing regional access to allogeneic stem cell transplantation and survival in Denmark in relation to family factors. While more research exists from the United States, differences in healthcare systems and population demographics limit direct comparison to Germany or Europe.

**Added value of this study**

This study provides a comprehensive, population-based analysis of AML and ALL in Germany to date, including 25,788 AML and 6480 ALL patients diagnosed between 2016 and 2021. By combining nationwide cancer registry data with area-based income and social deprivation indicators, we were able to describe incidence, age-specific survival, and socioeconomic disparities in outcomes. We demonstrate that survival of AML and ALL patients is highly age-dependent and provide treatment-specific survival data in Germany. Moreover, lower-income regions are associated with up to 4% reduced 3-year survival, highlighting the impact of socioeconomic disparities on outcomes in both leukaemia types. Finally, we provide projections of future case numbers, highlighting an expected 14.6% rise in AML cases and a 2.3% decline in ALL cases by 2050, offering essential information for healthcare planning, clinical trial design, and addressing regional disparities.

**Implications of all the available evidence**

Our findings highlight that AML and ALL survival in Germany is not only strongly age-dependent but also shaped by socioeconomic inequalities, with patients from lower-income regions experiencing worse outcomes. Current population-based survival data can inform guidelines and help evaluate new treatments while observed sex-specific survival differences warrant further investigation. Beyond treatment advances, efforts to reduce disparities should address broader structural determinants of health, including equitable access to specialized care and supportive resources.

## Introduction

Acute leukaemias are characterized by the rapid proliferation of immature hematopoietic cells stemming from lymphoid or myeloid progenitors. While acute leukaemias in paediatric patients are more often of lymphoblastic origin (ALL), elderly adults contend predominantly with acute myeloid leukaemia (AML).

Over the last decades, the management of acute leukaemias has changed significantly. Advances in molecular diagnostics and better understanding of disease biology have fostered the development of new treatment approaches with tyrosine kinase inhibitors (TKI), immunotherapies, or the BCL-2 inhibitor Venetoclax revolutionizing the management of acute leukaemias.^1^ Although age at diagnosis is still one of the most relevant prognostic factors, innovative therapies combined with better supportive care improved outcomes for patients with AML and ALL.^2^ Particularly in children with ALL, survival has increased dramatically, with 5-year OS rising from 10 to 25% in the 1950s to over 90% in recent years.^3^ In contrast, improvements of outcomes in elderly patients with AML or ALL remained limited, underscoring the challenges of treating older patients with acute leukaemias.^4^^,^^5^

Disease-specific registries for adult and childhood leukaemia provide detailed insights but often lack a population-based scope, which is necessary to support public health initiatives, including diagnostic services, treatment centres, and follow-up care programs.^6^ In contrast, population-based registries offer a comprehensive view of real-world outcomes, essential for refining clinical guidelines and targeted interventions.^7^ European epidemiological studies on acute leukaemia covering the United Kingdom or Denmark provided important insights in the management and outcomes of unselected patients with acute leukaemias.^8^^,^^9^ However, heterogeneity in demographics, socio-economic factors, and healthcare provision introduce considerable bias when these results are transferred to a different health care system.

Since comprehensive epidemiological studies on acute leukaemias in Germany are lacking, we analysed data from all German State Cancer Registries and the German Childhood Cancer Registry to investigate incidence, survival, treatment, and socioeconomic influences on acute leukaemias in Germany.

## Methods

### Study design

This study encompassed all regions of Germany by incorporating data from all 16 German federal states provided by the respective epidemiological and clinical state cancer registries, the German Centre for Cancer Registry Data, and the German Childhood Cancer Registry (GCCR). These registries organize and verify the reported data, track cancer incidence and trends, support epidemiological research, and help assess preventive and therapeutic measures. Treating physicians are required by law to report diagnoses, treatment changes, relapses, and deaths for adult patients to state cancer registries. Paediatric cancers (age 0–17 years) including leukaemias are predominantly reported to the GCCR and to a lesser extent to federal cancer registries. Although reporting to the GCCR is not mandatory in all federal states, all children with leukaemia in Germany are treated at specialized paediatric haematology centres that report to the registry, resulting in coverage of more than 95% of cases.

State registries annually report selected data to the German Centre for Cancer Registry Data and their survival data are harmonized with local population registers on a regular basis. This study analysed anonymized patient-level data as a cohort of cases from 2016 to 2021 based on ICD codes for AML (C92.0, C92.3, C92.5–C92.8, C93.0, C94.0, C94.2) and ALL (C91.0). Age-standardization was performed using the 2013 European Standard Population, with alternative standard populations also provided (Supplementary Table S1). Incidence and mortality rates were calculated based on data from the German Centre for Cancer Registry Data (age 18+) and the GCCR (age 0–17) and also included adult cases diagnosed via death certificates, while age- and therapy-related survival analyses were performed only using data directly provided by the state cancer registries derived from individual-level patient records. Data from 2021 were unavailable from five state cancer registries at the time of inquiry. Data privacy rules prohibited the publication of GCCR survival data, limiting reports for patients under 18 years to state cancer registries, which have a lower coverage of this age cohort. Average incidence rates were calculated as the 2016–2020 mean to account for potentially incomplete 2021 data. Mean mortality rates were estimated for years 2016–2020 based on the federal health reporting in Germany.^10^

Current and projected population data were sourced from the federal statistical office of Germany.^11^ Income data were obtained from the Economic and Social Science Institute of Germany.^12^ Socioeconomic influence on survival was estimated by correlation of survival rates of the German Centre for Cancer Registry Data with the 2019 per capita income of a patients county, a year in the middle of the analysis period and before the economically significant SARS-CoV-2 pandemic, and the German Index for Social Deprivation (GISD) provided by the Robert-Koch-Institute of the respective counties.^13^ In that year, counties had an average population of 207 398 people. Age and sex were included as covariates in a Cox proportional hazards model to adjust for related differences. Future annual leukaemia diagnoses were projected based on an official population estimate (Federal Statistical Office of Germany, Variant 1: Moderate development in fertility and life expectancy with low net migration).^11^ We applied the observed age-specific incidence rates (5-year age groups) of our study to the corresponding estimated population numbers of the year 2050.

This study was exempt from ethics approval as it used only anonymized or aggregated data.

### Statistical analysis

For descriptive statistics, age at diagnosis was summarized by median, first quartile (Q1), and third quartile (Q3), and compared using the Wilcoxon U-test. For survival analysis, we used the Kaplan–Meier estimator and the log-rank test. Follow-up time was calculated from the date of diagnosis until death or last known contact. Patients who were alive at the end of follow-up or lost to follow-up were censored at their last known alive date. Treatment outcomes were based on documented therapies, as information on treatment intent was not available. In patients with AML, treatments were categorized according to intensity. Intensive therapy referred to remission induction regimens administered to fit patients with curative intent, in line with current guidelines such as the ELN recommendations for AML. In contrast, lower-intensity therapy encompassed approaches typically used for unfit patients, including Venetoclax- and/or hypomethylating agent (HMA)-based regimens. Age groups were defined using current German guidelines. P-values <0.05 were considered significant, with Bonferroni correction applied (p_adj_ < 0.05) to correct for multiple testing when income and GISD groups or age groups in this context were compared. Uni- and multivariate analyses were used when outcomes were influenced by multiple factors, such as assessing income or GISD effects on survival while accounting for age and sex and results were reported as hazard ratios (HR) with confidence intervals (CI). The Gini coefficient was used to analyse the dispersion of the median incidence value distribution across counties. Age refers to diagnosis age in years, unless otherwise specified. All analyses were conducted using R version 4.4.2.

### Role of the funding source

The sponsors and funder (German Research Foundation) had no role in the study design, data handling, reporting, or publication decision.

## Results

This study included data from 32,268 patients aged 0–101 years diagnosed with AML or ALL in Germany between 2016 and 2021.

### Acute myeloid leukaemia

AML was diagnosed in 25,788 patients, which averages 4298 new AML cases per year including approximately 101 paediatric cases (<18 years) annually. The median age at diagnosis was 72.8 years (IQR 61.0–80.3), with 642 patients (2.5%) being under 19 years old (603 reported by GCCR). The 13,975 (54.2%) male patients were slightly younger than female patients, with a median age of 72.7 years (IQR 61.5–79.9) compared to 73.2 years (IQR 60.3–80.9; p = 0.00031).

### Incidence and mortality of AML in Germany

**Fig. 1:**
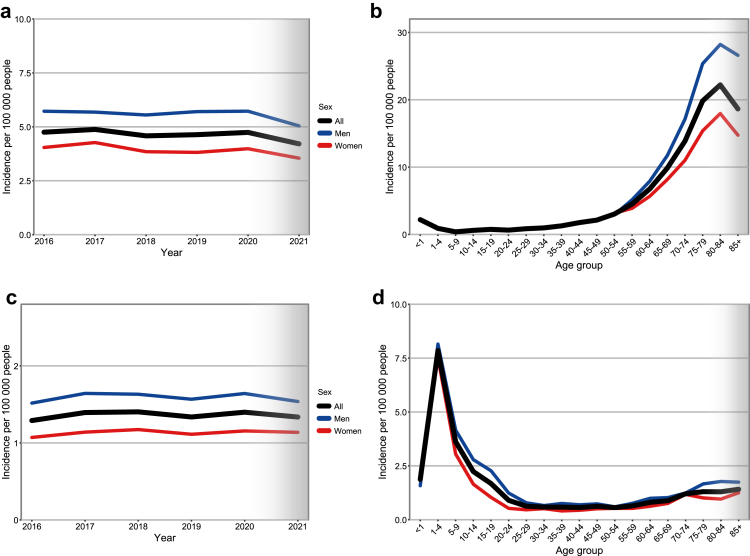
**Age-standardized and age-specific incidence rates for AML and ALL in Germany.** Age-standardized AML (a) and age-specific AML (b) and age-standardized ALL (c) and age-specific ALL (d) incidence rates per 100,000 in Germany. Black lines represent the total population, while blue and red lines show male and female rates, respectively. Men have higher incidence rates, especially in the elderly. Grey shading indicates years or age groups with potential missing diagnoses.

The mean age-standardized incidence rate (ASR) of AML was 4.72/100,000 (95% CI: 4.58–4.86) with a raw incidence rate of 5.26/100,000 (Fig. 1a, Supplementary Table S1 for different standard populations). ASR differed between men and women, with 5.68/100,000 for men and 3.99/100,000 for women, resulting in a male-to-female incidence ratio of 1.42. Age-specific incidence rates showed a small peak in children under 1 year (2.19/100,000) and remained similar between sexes up to age 54 (Fig. 1b). Incidence rates then rose sharply from age 55 onwards, with a steeper increase in men. The highest incidence was observed in the 80–84 age group (22.2/100,000), with rates of 28.2/100,000 for men compared to 18.0/100,000 for women. We observed an equal distribution of county-specific median AML incidence rates across Germany (Gini coefficient 0.14, Supplementary Figure S1a).

The mean age-standardized mortality rate was 4.40/100,000 with 4.92/100,000 for men and 3.92/100,000 for women, respectively.

### Survival of AML in Germany

Survival rates were calculated using data obtained from the state cancer registries and included 18,150 patients. Compared to the dataset of the German Centre for Cancer Registry Data, exclusions were mainly among those aged 60+ due to the omission of death certificate-only diagnoses. Median OS was 11.3 months (95% CI: 11.0–11.9) with a median follow-up time of 37.5 months (95% CI: 37.0–38.6). The respective 3-year and 5-year survival rates were 29.0% (95% CI: 28.3–29.7) and 23.8% (95% CI: 23.1–24.7) including all age groups and 28.5% (95% CI: 27.8–29.2) and 23.3% (95% CI: 22.6–24.2) for adult patients (Supplementary Figure S2).

**Fig. 2:**
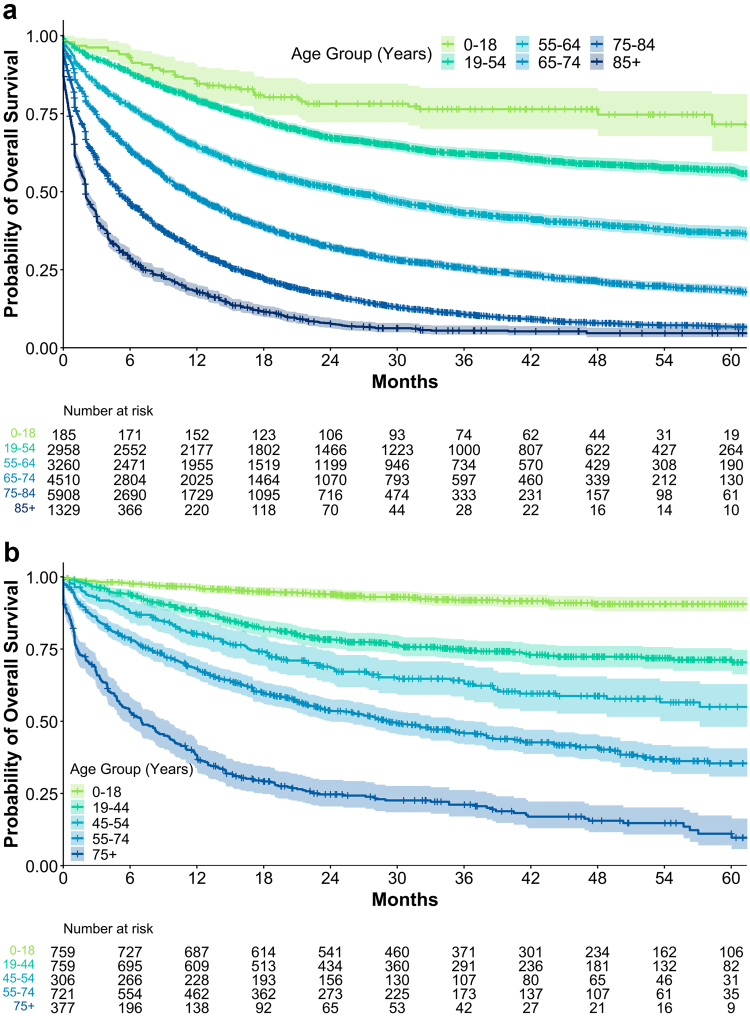
**Overall survival of patients with AML and ALL in Germany.** Overall survival of 18 150 patients with AML (a) and 2 924 patients with ALL (b) diagnosed between 2016 and 2021 in Germany, stratified by age groups.

Stratifying patients by age, survival was most favourable in patients with AML aged 0–18 years (5-year OS 71.6%) and lowest in those older than 85 years (5-year OS 4.7%, p < 0.0001, Tables 1 and 2, Fig. 2a). In general, the median OS was about 1.1 months shorter in men than in women (10.9 vs. 12.0 months, p = 0.00018, Supplementary Figure S3). This sex difference was not observed in patients ≤19 years and ≥65 years, but was pronounced in patients with AML aged 20–64 (Supplementary Figure S4).

**Table 1:** Survival and 60-day mortality rates in AML by age group.

Age group	Median OS (months, 95% CI)	3 y-OS % (95% CI)	5 y-OS % (95% CI)	60-day mortality % (95% CI)
0–18	Not reached	76.4 (70.2–83.2)	71.6 (63.0–81.3)	3.3 (0.7–5.8)
19–54	78.0 (78.0–NA)	62.1 (60.2–64.1)	57.0 (54.8–59.2)	5.8 (4.9–6.6)
55–64	25.4 (23.2–28.3)	43.2 (41.3–45.1)	36.8 (34.7–39.0)	11.5 (10.4–12.6)
65–74	11.1 (10.4–12.0)	25.5 (24.2–27.0)	18.3 (16.8–20.0)	19.5 (18.3–20.6)
75–84	5.0 (4.9–5.2)	10.8 (9.9–11.7)	6.8 (6.0–7.8)	30.8 (29.6–32.0)
85+	2.0 (2.0–2.2)	5.5 (4.2–7.1)	4.7 (3.4–6.4)	47.6 (44.8–50.2)

Median OS, survival rates at 3 and 5 years and early mortality rate of patients diagnosed with AML between 2016 and 2021 in Germany stratified by age. *Abbreviations: OS: Overall Survival, 3 y: 3-year, 5 y: 5-year, CI: Confidence Interval*.

Around 70% of patients were diagnosed with AML not otherwise specified (C92.0), while other ICD-coded subtypes were less common (Supplementary Figure S5a). To assess subtype-specific survival, we stratified patients by ICD-code (Supplementary Figure S5b). Outcomes were best for acute myelomonocytic leukaemia (C92.5) and impaired compared to AML not otherwise specified (C92.0) for acute erythroid (C94.0), megakaryocytic leukaemia (C94.2), and unspecified types (C92.7, Supplementary Table S2).

The 60-day mortality rate was 11.4% (95% CI: 10.8–12.0), ranging from 5.6% in patients under 54 years to 47.6% in those aged 85 years and older (Table 1).

**Fig. 3:**
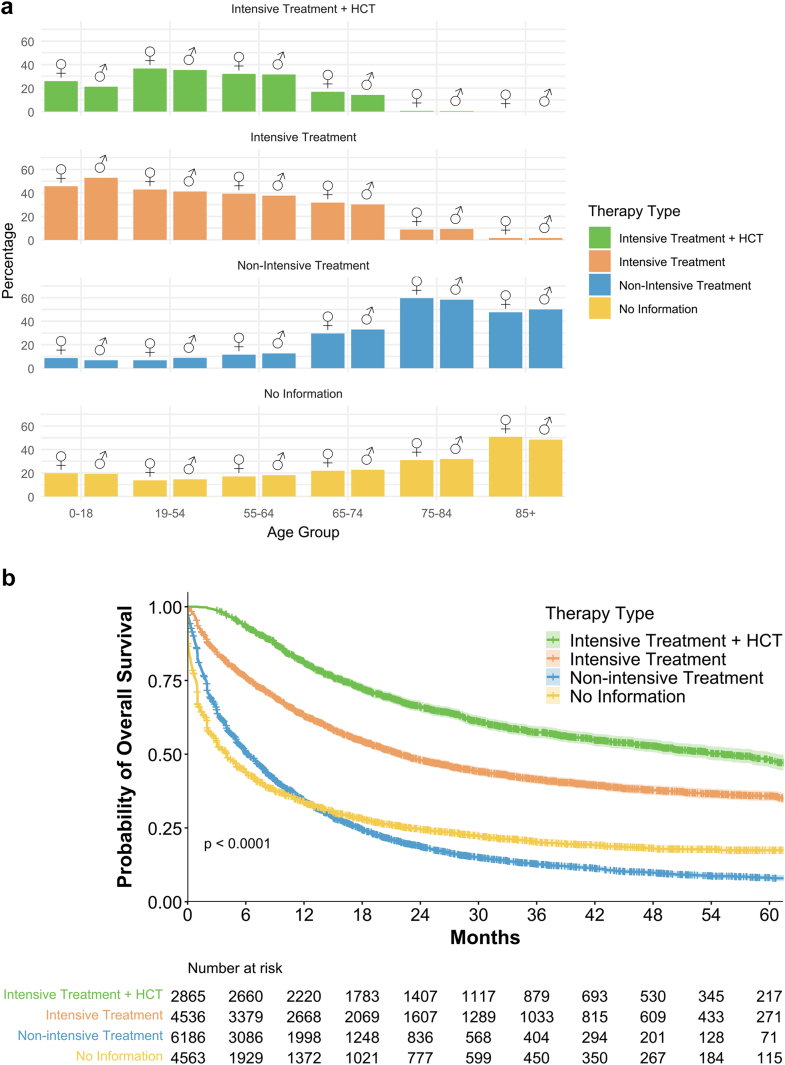
**Distribution of therapy types and overall survival in patients with AML.** (a) Distribution of therapy types by age and sex of 18 150 patients with AML (HCT: n = 2 865, Intensive Treatment: n = 4 536, Non-Intensive Treatment: n = 6 186, No Information: n = 4 563). Fewer than 10% of younger patients received non-intensive treatments, while over half of patients older than 75 years did. The highest rate intensive therapy with allogenic stem cell transplants (HCT) was in younger age groups. (b) Overall survival by therapy type across age groups. The highest survival rates were seen in patients receiving intensive treatment with HCT, while non-intensive treatments had the worst outcomes. *Abbreviations: HCT: allogeneic hematopoietic stem cell transplantation*.

Of the 18,150 patients with AML included in the survival analysis, 13 587 had documented information about applied treatments. Among these, 6186 (45.5%) patients received non-intensive therapies, 4536 (33.4%) received intensive treatment (anthracycline-based or similar induction regimens) without allogeneic hematopoietic stem cell transplantation (HCT) and 2865 (21.1%) received intensive treatment including HCT. Non-intensive treatments were uncommon in young patients but frequently used in elderly (Fig. 3a). The highest median OS with 55.3 months (95% CI: 49.1–60.9) was observed in patients receiving intensive treatment including HCT (Fig. 3b). Intensively treated patients without HCT had a significantly lower median OS of 22.0 months (95% CI: 20.6–23.6, p < 0.0001). Median OS was lowest in patients with no documented treatment (4.0 months, 95% CI: 3.6–4.2, n = 4563), followed by those receiving non-intensive treatment (6.1 months, 95% CI: 6.0–6.7). An association between intensive treatment and better outcomes was seen across all ages (Supplementary Figure S6).

In Germany, Venetoclax was approved in 2021 for treating patients with AML ineligible for intensive chemotherapy. However, Venetoclax-based treatments were already used off-label before 2021. Of the 6186 non-intensively treated patients with AML, 717 (11.6%) received Venetoclax-based regimens, including 506 aged 75 years or older. These 506 patients [median age: 79 years (IQR 77–82), 54.0% male] showed a significantly longer survival (median OS: 14.0 months; 95% CI: 11.9–15.3) compared to patients ≥75 years with AML treated with non-intensive [n = 3 622, median age: 80 years (IQR 78–83), median OS: 4.8 months; 95% CI: 4.2–5.0] or intensive regimens [n = 586, median age: 77 years (IQR 76–80), median OS: 8.1 months; 95% CI: 7.3–9.0, p < 0.0001; Supplementary Figure S7].

### Acute lymphoblastic leukaemia

Between 2016 and 2021, we identified 6480 patients diagnosed with ALL in Germany, representing an average of 1080 new cases annually, with approximately 520 cases in patients under 18 years per year. Of these patients, 3759 (58.0%) were male and 3201 (49.4%) were younger than 19 years (3117 reported by GCCR). Median age was 19.4 years (IQR 5.2–58.6) with no significant difference between male (19.4 years, IQR 5.7–56.2) and female patients (19.8 years, IQR 4.5–62.0, p = 0.69). Information on lineage assignment was available for 1 904 of 3355 adults (>18 years), indicating 1381 B-ALL and 523 T-ALL cases.

**Table 2:** Survival and 60-day mortality rates in ALL by age group.

Age group	Median OS (months, 95% CI)	3 y-OS % (95% CI)	5 y-OS % (95% CI)	60-day mortality % (95% CI)
0–18	Not reached	91.9 (89.8–94.1)	90.6 (88.2–93.1)	1.1 (0.3–1.8)
19–44	Not reached	74.7 (71.4–78.2)	71.3 (67.5–75.3)	2.2 (1.2–3.3)
45–54	Not reached	63.5 (57.9–69.7)	55.0 (48.1–62.8)	5.2 (2.7–7.7)
55–74	29.3 (24.0–37.0)	45.8 (42.0–50.1)	35.4 (30.9–40.6)	10.7 (8.4–13.0)
75+	7.0 (5.4–9.5)	21.1 (17.0–26.2)	11.1 (7.0–17.4)	27.7 (23.0–32.1)

Median OS, survival rates at 3 and 5 years and early mortality rate of patients diagnosed with ALL between 2016 and 2021 in Germany stratified by age. *Abbreviations: OS: Overall Survival, 3 y: 3-year, 5 y: 5-year, CI: Confidence Interval*.

### Incidence and mortality of ALL in Germany

The mean ASR in Germany for ALL was 1.36/100,000 (95% CI: 1.30–1.43), with a raw incidence of 1.30/100,000 (Fig. 1C, Supplementary Table S1). ASR differed significantly by sex with 1.60/100,000 in men and 1.13/100,000 in women, resulting in a male-to-female ratio of 1.40.

The age-specific incidence rate for ALL peaked at 7.85/100,000 in children aged 1–4 years, with the lowest rates observed in those between 25 and 59 years of age (Fig. 1D). Incidence rates steadily increased after the age of 60 years, reaching a rate of 1.41/100,000 in those older than 85 years. We observed an equal distribution of ALL-cases across Germany when comparing county-specific median incidence rates (Gini coefficient 0.25, Supplementary Figure S1b).

The mean age-standardized mortality rate was 0.38/100,000 inhabitants overall, 0.47/100,000 in men and 0.30/100,000 in women, respectively.

### Survival of all in Germany

Survival rates were calculated using data obtained from the state cancer registries, covering 2922 patients with ALL, excluding GCCR data and 431 adults listed only in the German Cancer Registry Centre dataset. For the entire cohort (n = 2922), including both paediatric (n = 758) and adult patients (n = 2164), the 3-year OS rate was 64.0% (95% CI: 62.2–65.9), and the 5-year OS rate was 57.9% (95% CI: 55.7–60.2). Among adult patients only, the 3-year and 5-year OS rates were 54.1% (95% CI: 51.9–56.5) and 46.4% (95% CI: 43.7–49.2), respectively. When stratified by age, median OS was not reached in the 0–18, 19–44, and 45–54 age groups, while median OS was 29.3 months (95% CI: 24.0–37.0) for those aged 55–74 and 7.0 months (95% CI: 5.4–9.5) for those 75 years or older (p < 0.0001, Fig. 2b, Table 2). Significant sex-dependent differences in OS were seen in patients aged 55–74 years, with a 3-year OS of 41.4% (95% CI: 36.3–47.2) in male compared to 51.3% (95% CI: 45.6–57.7) in female patients (p = 0.0099, Supplementary Figure S8, Supplementary Table S3).

Due to differences in treatment protocols and outcomes between paediatric and adult patients with ALL, we focused on adult patients for treatment-related outcomes. Among 2164 adult patients, 1222 had B-ALL, 289 had T-ALL, and 653 lacked lineage information. Therapy data were available for 1798 patients, including 362 who underwent HCT. Among 1083 B-ALL patients with information on applied therapies, 292 received a TKI-containing combination therapy, suggestive of a Philadelphia-chromosome positive ALL. Survival in the TKI-treated ALL patients was comparable to non-TKI-treated patients across all ages (18–54 years: 3-year OS 75.2% vs. 69.5%, p = 0.16; 55+ years: 3-year OS 42.2% vs. 42.5%, p = 0.42, Supplementary Figure S9). Three-year OS after intensive treatment with HCT was similar in patients aged 19–44 and 45–54 (75.0% vs. 77.3%, p = 0.91), but dropped to 52.5% in those aged 55–74 (p = 0.00014, Supplementary Figure S10).

The 60-day mortality rate of patients with ALL older than 18 years was 9.9% (95% CI: 8.6–11.2%), with rates of 2.3% in those aged 18–54 years, rising to 17.0% in ages 55–74 and 27.3% in those older than 75 years (Table 2).

### Impact of socioeconomic differences on survival in acute leukaemia

**Fig. 4:**
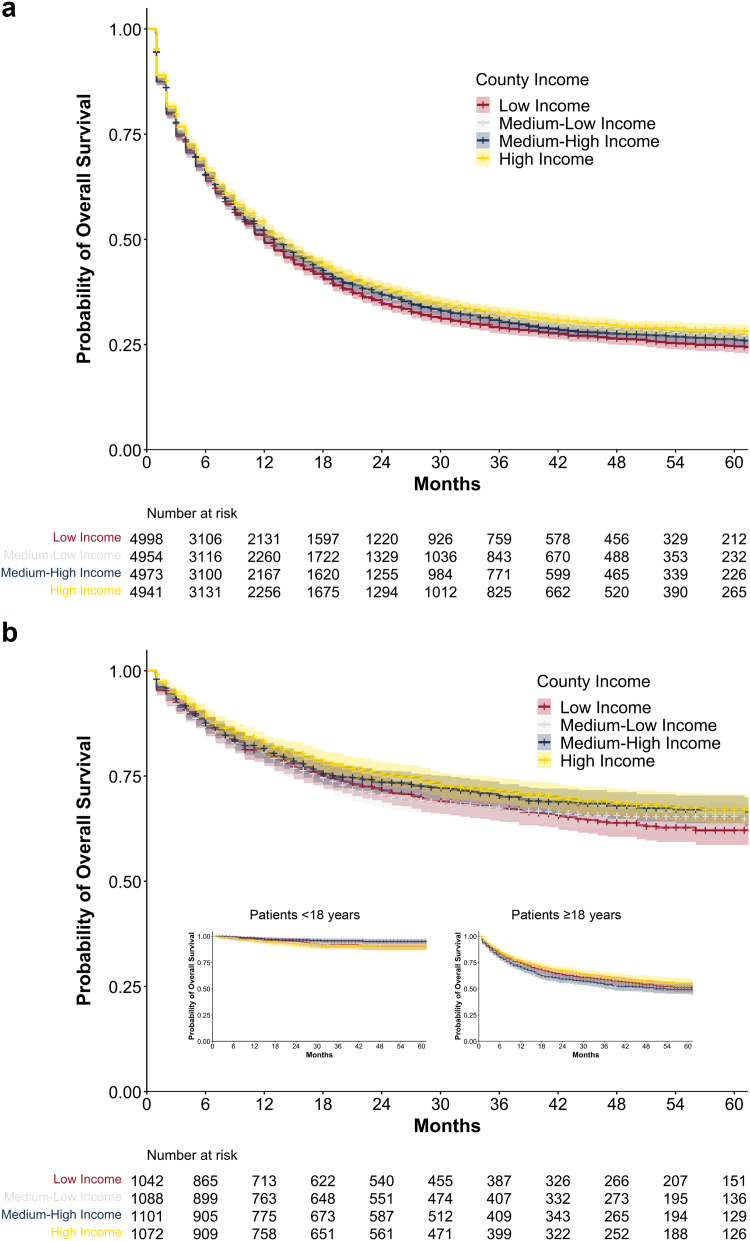
**AML and ALL survival rates by county per capita income.** Overall survival of patients with AML (a) and ALL (b) stratified by county per capita income. The 3-year survival rates were 29.1% (95% CI: 27.7–30.6) in low-income counties (mean income 20,528 EUR/year) and 32.5% (95% CI: 31.0–34.0) in high-income counties (mean income 27 047 EUR/year) in AML and 71.5% (95% CI: 68.6–74.6) in high-income counties compared to 67.3% (95% CI: 64.3–70.6) in low-income counties in ALL. Likely due to small numbers, this was not seen in ALL when splitting the cohort at 18 years (<18 years n = 1398, ≥18 years n = 2905, small plots).

After grouping patients into income quartiles based on their mean county income, median OS in patients with AML was 14.0 months (95% CI: 13.0–15.0) in the wealthiest income group with a mean income of 27,047 EUR/year (SD 2447 EUR), compared to 12.0 months (95% CI: 11.0–13.0) in the poorest income group with a mean income of 20 528 EUR/year (SD 936 EUR, p_adj_ = 0.0013, Fig. 4a). Accordingly, 3-year OS rates were 32.5% (95% CI: 31.0–34.0) and 29.1% (95% CI: 27.7–30.6) with a median age of 70.4 and 71.3 years (p = 0.00074), respectively. Patients with ALL in the highest income group were significantly younger than those in the lowest income group (median age: 38.2 years vs. 48.8 years, p < 0.0001) and had a higher 3-year OS rate [71.5% (95% CI: 68.6–74.6) vs. 67.3% (95% CI: 64.3–70.6), p = 0.0032, Fig. 4b]. To assess the impact of income on survival, univariate and multivariate Cox models were conducted, adjusting for age, sex, AML subtype by ICD code, and income. In AML, higher income was linked to lower mortality in univariate analysis (HR 0.91, 95% CI: 0.87–0.96, p < 0.00034), but this was not significant after adjustment in the multivariate analysis (HR 0.96, 95% CI: 0.91–1.01, p = 0.11; Supplementary Table S4). Similarly, in ALL the highest income group showed a survival advantage in univariate analysis (HR 0.79, 95% CI: 0.69–0.90, p < 0.00055), which was not significant in multivariate analysis (HR 0.96, 95% CI: 0.84–1.09, p = 0.52; Supplementary Table S5). Likewise, no correlation was observed between survival and income when the cohort was split at age 18 (Fig. 4b).

To account for factors beyond income, we extended our analysis using the GISD, a deprivation index based on education, employment, and income, weighted through principal component analysis to assess regional health disparities. Similarly, we grouped patients into GISD quartiles. In AML, survival analysis by GISD revealed a longer median OS of 14.0 months (95% CI: 13.9–15.0) in the least deprived group and a shorter median OS of 12.9 months (95% CI: 12.0–13.0) in the most deprived group (p_adj_ < 0.0028) while age was similar between these groups (70.3 vs. 70.3 years, p = 0.19). Both univariate and multivariate Cox regression analyses showed that higher levels of social deprivation were significantly associated with increased mortality, independent of other factors such as age at diagnosis, sex, and leukaemia subtype (Supplementary Table S6).

We examined whether the lower OS in men with AML varied across GISD groups by stratifying by age and sex. No sex-related differences were seen in patients ≤40 years, while among men aged 40–59 years those in more deprived counties had worse survival than those in less deprived counties (3-year OS: 50.1% (95% CI: 45.4–55.2) vs. 57.0% (95% CI: 52.3–62.2), p_adj_ = 0.035). No significant differences were observed in the other age- and sex-stratified GISD groups.

Similarly in ALL, the least deprived group had a higher 3-year OS of 72.7% (95% CI: 69.8–75.7), compared to 65.7% (95% CI: 62.7–69.0) in the most deprived group (p_adj_ = 0.0013). However, the least deprived group was younger (30.6 vs. 44.4 years, p < 0.0001). In both univariate and multivariate Cox regression analyses, higher social deprivation showed a trend toward increased mortality, with significant associations in univariate models that attenuated after adjustment for age at diagnosis and sex (Supplementary Table S7). Similar results were observed when the cohort was stratified at age 18.

### Projection of acute leukaemia cases in Germany for the year 2050

Like many Western countries, Germany has an aging population, and diseases common in the elderly are expected to rise. Based on population estimates of the German federal statistical office, we project a 14.6% increase in new AML diagnoses over the next 25 years, from 4298 to 4925 new cases annually by 2050. In ALL, we calculated a slight decrease of −2.3% new diagnoses over the next 25 years, from 1080 cases annually to 1052 by 2050.

## Discussion

We provide a comprehensive overview of epidemiology and outcomes of acute leukaemia in Germany by analysing over 32,000 patients diagnosed between 2016 and 2021. The ASR of 4.72/100,000 for AML is slightly higher than reported for other northern European countries (2.9–4.1/100,000) but lower than in USA (German data transferred to US2000 standard population 3.51/100,000 vs. 4.3/100,000 for USA).^14^^,^^15^ Noted deviation may reflect differences in ICD coding practices, differences in healthcare systems and diagnostic reporting, or different prevalence of AML risk factors in the population such as prior therapies, smoking, or obesity.

The observed 5-year OS rates for AML (23.8% overall, 23.3% > 18 years) and ALL (57.9% overall, 46.4% > 18 years) are consistent with other studies despite variations in databases and populations.^5^^,^^16^ The population-based EUROCARE-6 study found 5-year AML survival rates of 20.6%–26.1% in European countries with health spending levels similar to Germany.^17^ The US-based SEER database reports a 5-year OS rate of 28% in AML with a lower median age of 65 years compared to a median age of 72.8 years in our analysis.^4^

Intensive treatment regimens provide the best chances for long-term survival especially in younger patients. Similar to a Swedish study where 91–98% of patients were considered eligible for intensive therapy, over 90% of patients aged 19–64 received intensive treatment when unknown treatments were excluded in our analysis.^18^ A recent study from the UK reported 5-year OS rates of 53.9% in younger patients with AML aged 18–54 years eligible for intensive treatment which is comparable to the 5-year OS rate of 57.0% in our analysis for the same age group.^8^

The introduction of Venetoclax has changed the therapeutic landscape of AML significantly and became the new standard of care for patients ineligible for intensive therapy. Importantly, the more favourable toxicity profile and high efficacy of venetoclax in combination with HMA appears to be advantageous and does not necessarily preclude further, more intensive treatments. In this population-based analysis, patients ≥75 years treated with Venetoclax-based non-intensive therapies had a better OS than those receiving intensive or non-intensive treatments without Venetoclax. The median OS of 14.0 months in these Venetoclax-treated patients matches the outcome data from the VIALE-A trial but surpasses other real-world data, where median OS mostly ranges between 8 and 12 months.^19^

ALL incidence rates aligned well with other Western countries. The reported ASR for Germany closely matched the SEER-reported 1.7–1.9/100,000 for the same period and are well in range with figures reported internationally ranging from 1.08 to 2.12/100,000.^20^

We noted slightly lower ALL survival rates in our cohort of 2164 adult patients (5-year OS: 46.4%, median age 55 years) compared to those published for 127 adult Israeli patients (median age 44 years) treated under the GMALL 07/2003 protocol between 2007 and 2020 with a 5-year OS of 57%.^21^ The 3-year OS of 75.2% in TKI-treated and therefore potentially Philadelphia-positive B-ALL patients aged 18–55 in our cohort was similar to the 76% 3-year OS in 174 Philadelphia-positive B-ALL patients reported by GMALL between 2016 and 2022.^22^ In line with other analyses, we observed similar outcomes in TKI-treated and non-TKI-treated B-ALL patients after age-matching to control for increased Philadelphia-positivity in older patients.^23^ Interestingly, we observed a 10% higher 3-year OS in women than men in patients aged 55–74 years. While better survival in women with malignancies has been observed by others and was also seen in AML in this study, in ALL this has been predominantly described in children.^24^

We observed a HCT rate of 41.6% for patients with AML after excluding missing therapy data, which is similar to the 38% reported in a Swedish registry study of adult AML patients under 60 years.^25^ Comparable to published 5-year OS rates of 30–55% depending on cohort and criteria, we noted 3-year and 5-year OS of 57.4% and 48.1% for HCT recipients.^26^ For ALL, post-HCT outcomes were similar to published data, though cross-country comparisons are limited by treatment variability.^27^

The German healthcare system provides full coverage for leukaemia treatment, regardless of income, insurance or social status of patients. However, we found a potential association between survival and the mean per capita income of a patient's county. Although the effect was not significant in the multivariate analysis, this potential association merits further investigation, especially since similar patterns have been observed for other cancers in countries such as the USA and the UK, as well as in a small German study focusing on patients with leukaemia.^8^^,^^28^ Furthermore, low-income patients are more likely to show unfavourable genetic patterns, disadvantageous epigenetic changes and less complete remission after induction chemotherapy while potentially presenting with increased inflammatory activity.^29^ When incorporating additional deprivation factors such as education and employment through the GISD, social deprivation had a greater impact on survival in AML than in ALL. Unhealthy behaviours associated with lower socioeconomic status, such as smoking, poor diet, and obesity, may play a larger role in AML outcomes since patients are typically older.

Based on projected demographic changes, we estimate a 14.6% rise in new diagnoses of AML by 2050 compared to 2020, potentially increasing the raw incidence rate to 6.1/100,000. This aligns with projections for other malignancies in Germany and the rest of the world.^30^ The proportion of people over 65 years in Germany will rise from 18.3 million in 2020 to 23.1 million, likely driving an increase in age-related diseases like AML.^11^ Although AML is a rare entity, its treatment is expensive and requires specific resources, including accredited stem cell facilities. Therefore, accurate projections of new cases per year for resource-intensive diseases like acute leukaemias are essential for efficiently allocating potentially limited financial and personnel resources.

Cancer registries predominantly track incidences, explaining partly missing disease and therapy data or information about comorbidities. These limitations hindered reporting more detailed information about molecular classifications, prognostic scores, and sequential treatment algorithms applied. Furthermore, information about the completeness of data for individual patients is lacking. HCT survival data should be interpreted cautiously given potential immortality bias. This study reports net overall survival rather than relative survival, thereby potentially underestimating leukaemia-related outcomes. Survival rates in patients <20 years might be inaccurate due to missing GCCR survival data. Expanding data linkages to genetic labs, comorbidities, and demographic factors would enhance the analysis and scope. Since income and GISD data are available only at aggregate levels, we could not correlate them with treatments and thus cannot account for treatment variations by income or GISD. While we trust the documented treatments are valid, missing data in some cases may introduce bias in treatment-related survival analysis.

This is the first population-based analysis of acute leukaemia treatment and survival in Western Europe's most populous country with 83 million inhabitants. Analysing 25,788 AML and 6480 ALL cases, we present a comprehensive overview of leukaemia epidemiology and survival in Germany with incidence rates comparable to other large western countries. While survival is high in young patients, further improvements in treatments are needed for elderly considering growing patient numbers. Leveraging cancer registries to uncover socioeconomic disparities and real-world outcomes provides one of several options to address these future challenges.

## Contributors

David Baden and Claudia D. Baldus conceptualized the study. Data curation was performed by David Baden and Nadine Wolgast, and formal analysis by David Baden, Nadine Wolgast, and Philipp M. Altrock. David Baden, Nadine Wolgast, Claudia D. Baldus, Jakob Voran and Philipp M. Altrock had access to raw data and verified the data. Investigation and project administration were led by David Baden and Claudia D. Baldus, with supervision by Lars Fransecky, Alexander Katalinic, and Claudia D. Baldus. Visualization was done by David Baden and Jakob Voran. The original draft was written by David Baden and Claudia D. Baldus, and review and editing were contributed by all authors. All authors interpreted the data, verified the results, approved the final manuscript, and are accountable for all aspects of the work.

## Data sharing statement

The data sets analysed in this study were provided by the 16 German state cancer registries, the German Cancer Registry Centre, and the German Childhood Cancer Registry and are available to researchers upon reasonable request at the respective registries.

## Editor note

The Lancet Group takes a neutral position with respect to territorial claims in published maps and institutional affiliations.

## Declaration of interests

The authors declare no relevant financial or non-financial interests regarding data and conclusions presented in this publication. Philipp M Altrock received support from KITE Pharma and CRISPR Therapeutics, Jakob Voran received support from AstraZeneca GmbH, DGK (Deutsche Gesellschaft für Kardiologie) and DZHK (Deutsche Zentrum für Herzkreislaufkrankheiten) and Lilly. Manuel Hecht received support by the Federal Ministry of Research, Technology and Space of Germany. Gunnar Cario received support by Amgen, Clinigen, Jazz, and Servier. Christoph Röllig received support by AbbVie, Astellas, Bristol Myers Squibb, Daiichi Sankyo, Janssen, Jazz, Novartis, Otsuka, Pfizer, and Servier. Claudia D Baldus received support by Johnson & Johnsen, Autolus, and Astellas.
