# Supplementary material for: Epidemiology, survival, and treatment of acute myeloid and lymphoblastic leukaemia in Germany: a nationwide population-based registry analysis
Source: Lancet Reg Health Eur. 2025 Oct 18;59:101503. doi: 10.1016/j.lanepe.2025.101503 (PMC12556320; doi:10.1016/j.lanepe.2025.101503)
Supplement: Supplement [file mmc1.pdf]

Supplement - Epidemiology, survival, and treatment of acute myeloid and lymphoblastic leukaemia in Germany: a nationwide population-based registry analysis

David Baden[1,2#], Nadine Wolgast[1,2,3], Philipp M Altrock[1,3,4], Sophie Steinhäuser[1,2], Jakob Voran[5], Thomas Beder[1,2], Manuel Hecht[1,2], Cornelia Baden[1,2], Lorenz Bastian[1,2,3], Cécile Ronckers[6], Jacqueline Müller-Nordhorn[7], Soo-Zin Kim-Wanner[8], Martin Neumann[1,2,3], Lars Fransecky[1,2], Gunnar Cario[2,3,9], Christoph Röllig[10], Alexander Katalinic[2,11], Claudia D Baldus[1,2,3]

[1] *Department of Medicine II, Haematology and Oncology, University Hospital Schleswig-Holstein, Christian-Albrechts-University, Kiel, Germany*

[2] *University Cancer Centre Schleswig-Holstein, University Hospital Schleswig-Holstein, Kiel, Germany*

[3] *Clinical Research Unit CATCH ALL (KFO 5010), Kiel, Germany*

[4] *Department of Theoretical Biology, Max Planck Institute for Evolutionary Biology, Ploen, Schleswig-Holstein, Germany*

[5] *Department of Internal Medicine III, Cardiology and Intensive care, University Hospital Schleswig-Holstein, Kiel, Germany*

[6] *Division of Childhood Cancer Epidemiology, German Childhood Cancer Registry, Institute for Medical Biostatistics, Epidemiology and Informatics, University Medical Centre of the Johannes Gutenberg University, Main, Germany*

[7] *Bavarian Cancer Registry, Bavarian Health and Food Safety Authority, Nuremberg, Germany*

[8] *Hessian Cancer Registry, Hessian Office for Health and Care, Frankfurt, Germany*

[9] *Department of Paediatrics I, ALL-BFM Study Group, Christian-Albrechts University Kiel and University Medical Centre Schleswig-Holstein, Kiel, Germany*

[10] *Medical Department I, University Hospital of TU Dresden, Dresden, Germany*

[11] *Institute for Social Medicine and Epidemiology, University Hospital Schleswig-Holstein, Christian-Albrechts-University, Lübeck, Germany*

[#] *Corresponding author*

Table of content

page	content
**3**	Supplement Figure 1: Incidence rate of AML and ALL per county in Germany
**4**	Supplement Figure 2: Overall survival of adult patients with AML and ALL
**4**	Supplement Figure 3: Overall survival of patients with AML stratified by sex
**5**	Supplement Figure 4: Overall survival of patients with AML by age and sex
**6**	Supplement Figure 5: Distribution and overall survival of patients with AML by AML-type
**7**	Supplement Figure 6: Overall survival of patients with AML by therapy type and age groups
**8**	Supplement Figure 7: Treatments in patients with AML ≥75 years
**9**	Supplement Figure 8: Overall survival of patients with ALL by age and sex
**10**	Supplement Figure 9: Overall survival of patients with B-ALL by age group and TKI-treatment status
**11**	Supplement Figure 10: Overall survival of patients with ALL undergoing allogeneic hematopoietic stem cell transplantation by age group
**12**	Supplement Table 1: Raw and age-standardized incidence rates by standard population
**13**	Supplement Table 2: Uni- and multivariate analysis of survival in AML
**13**	Supplement Table 3: Uni- and multivariate analysis of survival in ALL
**14**	Supplement Table 4: Uni- and multivariate analysis of income-related survival in AML
**14**	Supplement Table 5: Uni- and multivariate analysis of income-related survival in ALL
**15**	Supplement Table 6: Uni- and multivariate analysis of GISD-related survival in AML
**15**	Supplement Table 7: Uni- and multivariate analysis of GISD-related survival in ALL

Supplement Figure 1: Incidence rate of AML and ALL per county in Germany

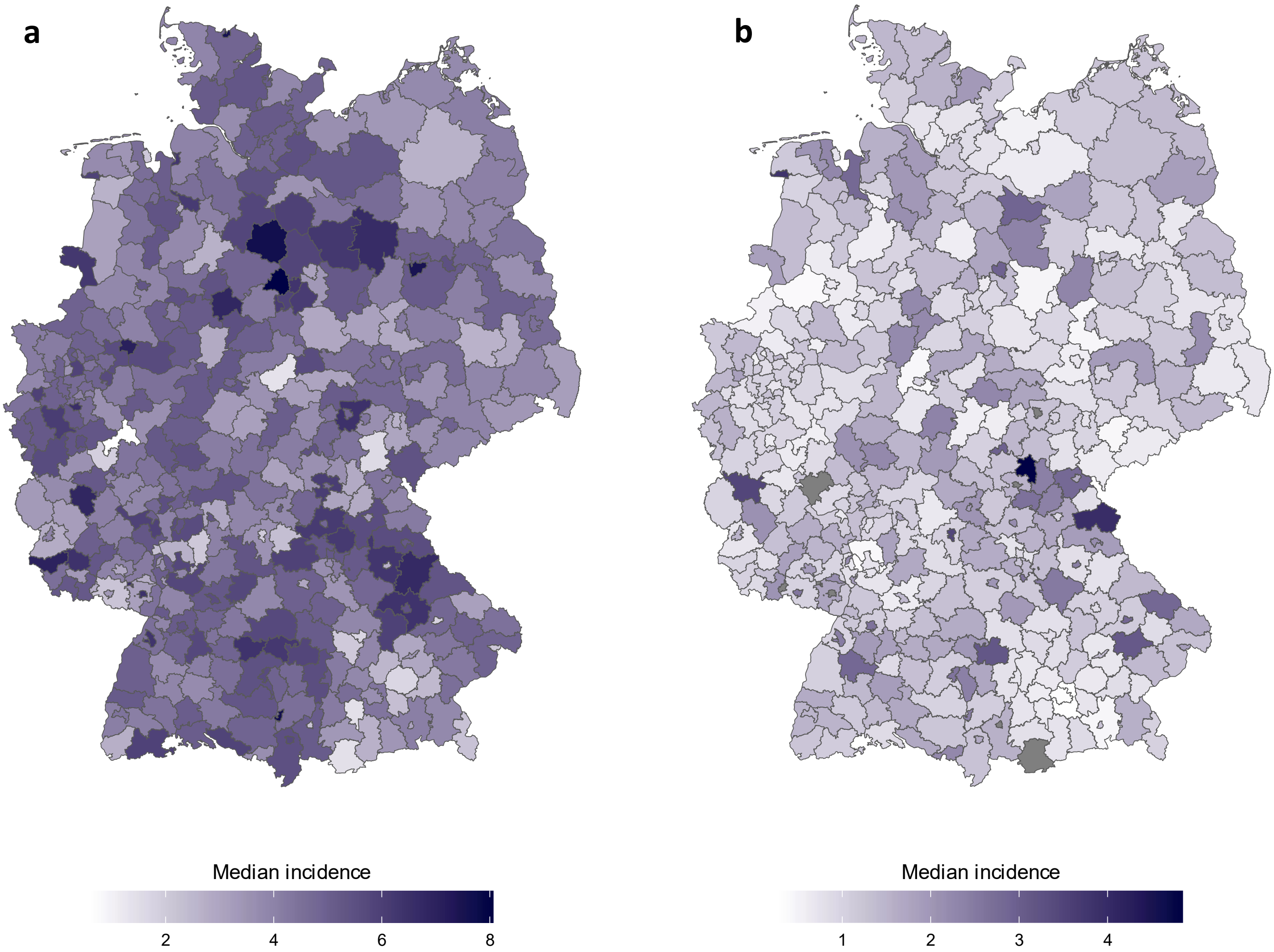

Supplement Figure 1: Median incidence rate per 100 000 over 5 years (2016-2020) of AML (a) and ALL (b) per county in Germany. AML analysis included 25 541 patients, excluding 247 from the Children's Cancer Registry due to missing county data. ALL analysis included 4 833 patients, excluding 1 647 children due to the same limitation.

Supplement Figure 2: Overall survival of adult patients with AML and ALL

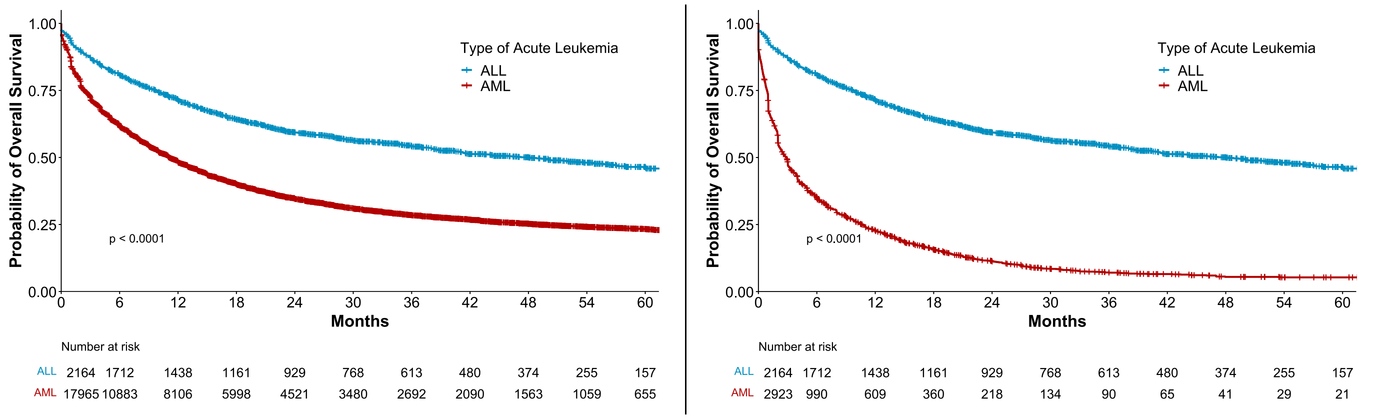

Supplement Figure 2: Unmatched (left figure) and matched (right figure) comparison of overall survival between adult patients with AML and ALL in Germany. Patients were matched by age and sex to control for differences in these variables between AML and ALL.

Supplement Figure 3: Overall survival of patients with AML stratified by sex

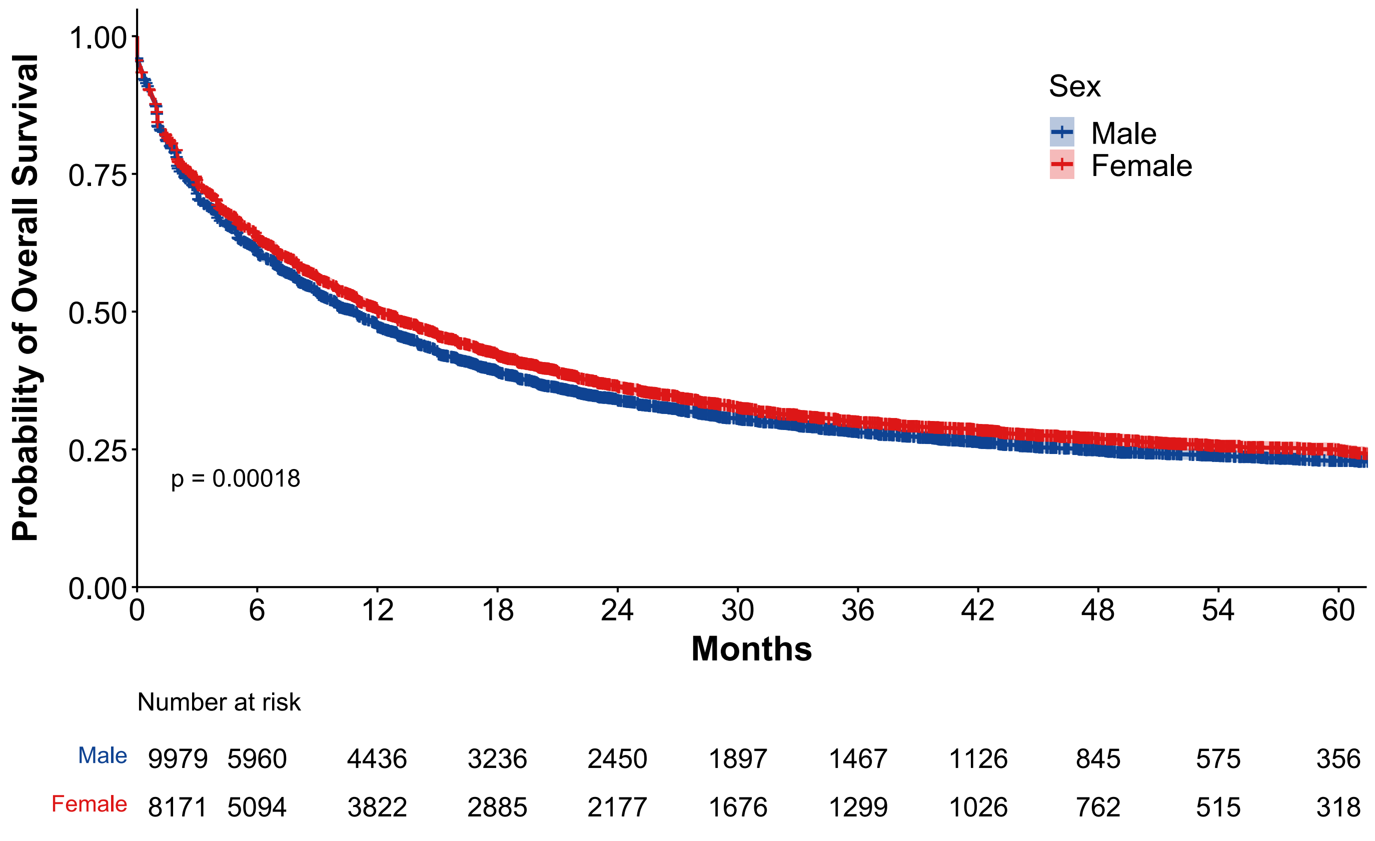

Supplement Figure 3: Overall survival of male (blue) and female (red) patients with AML over all ages. At three years, overall survival was 28·2% for men and 30·0% for women. The 5-year OS rate is 22·9% for males and 24·9% for females, showing a marginally better survival for female patients (p=0·00018).

Supplement Figure 4: Overall survival of patients with AML by age and sex

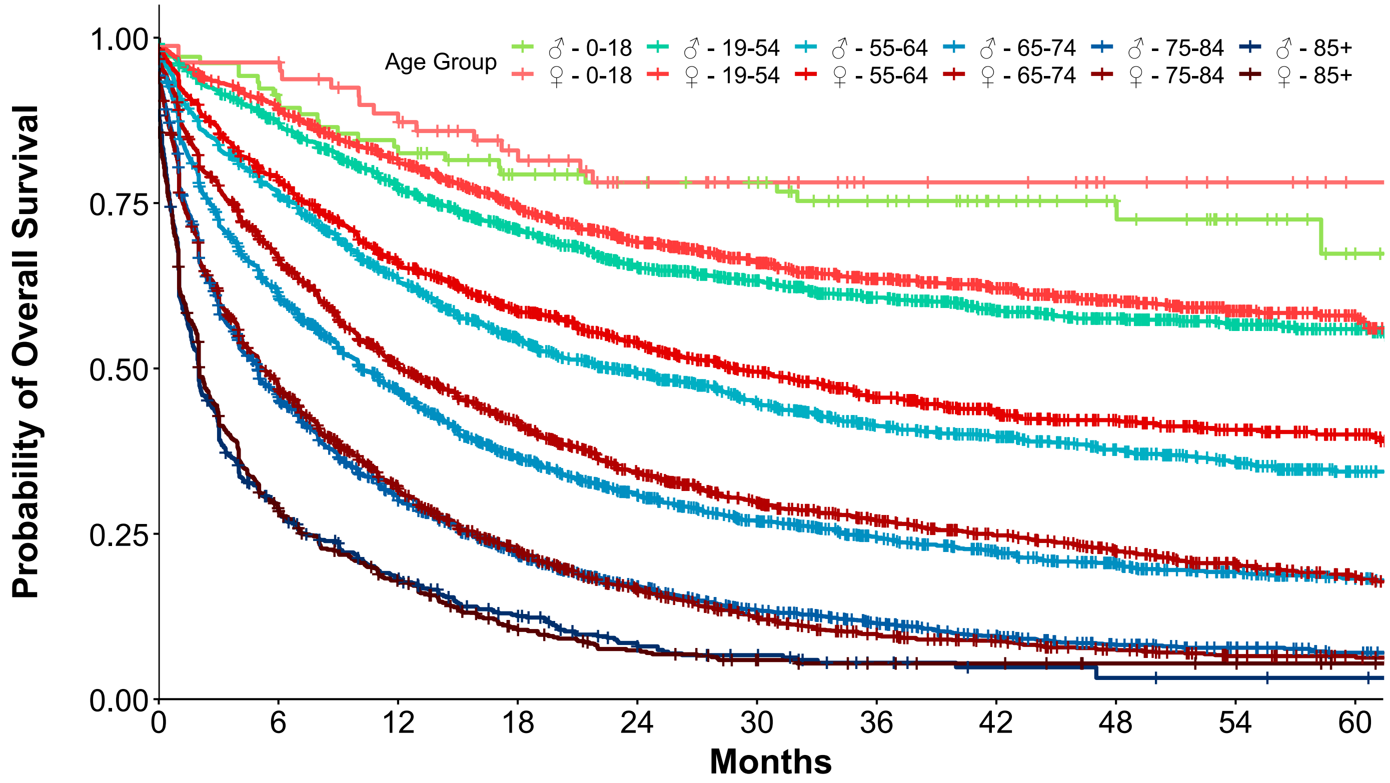

Number at risk

	0	6	12	18	24	30	36	42	48	54	60
♂ - 0-18	104	94	84	69	63	59	47	36	27	19	11
♀ - 0-18	81	77	68	54	43	34	27	26	17	12	8
♂ - 19-54	1480	1258	1062	875	714	600	502	401	311	211	137
♀ - 19-54	1478	1294	1115	927	752	623	498	406	311	216	127
♂ - 55-64	1877	1405	1107	837	656	516	390	303	233	170	99
♀ - 55-64	1383	1066	848	682	543	430	344	267	196	138	91
♂ - 65-74	2582	1550	1127	790	579	429	320	247	182	116	74
♀ - 65-74	1928	1254	898	674	491	364	277	213	157	96	56
♂ - 75-84	3298	1479	949	605	405	273	198	133	88	56	33
♀ - 75-84	2610	1211	780	490	311	201	135	98	69	42	28
♂ - 85+	638	174	107	60	33	20	10	6	4	3	2
♀ - 85+	691	192	113	58	37	24	18	16	12	11	8

Supplement Figure 4: Overall survival of male (green/blue) and female (red) patients with AML in Germany diagnosed between 2016 and 2021 per age group.

Supplement Figure 5: Distribution and overall survival of patients with AML by AML-type

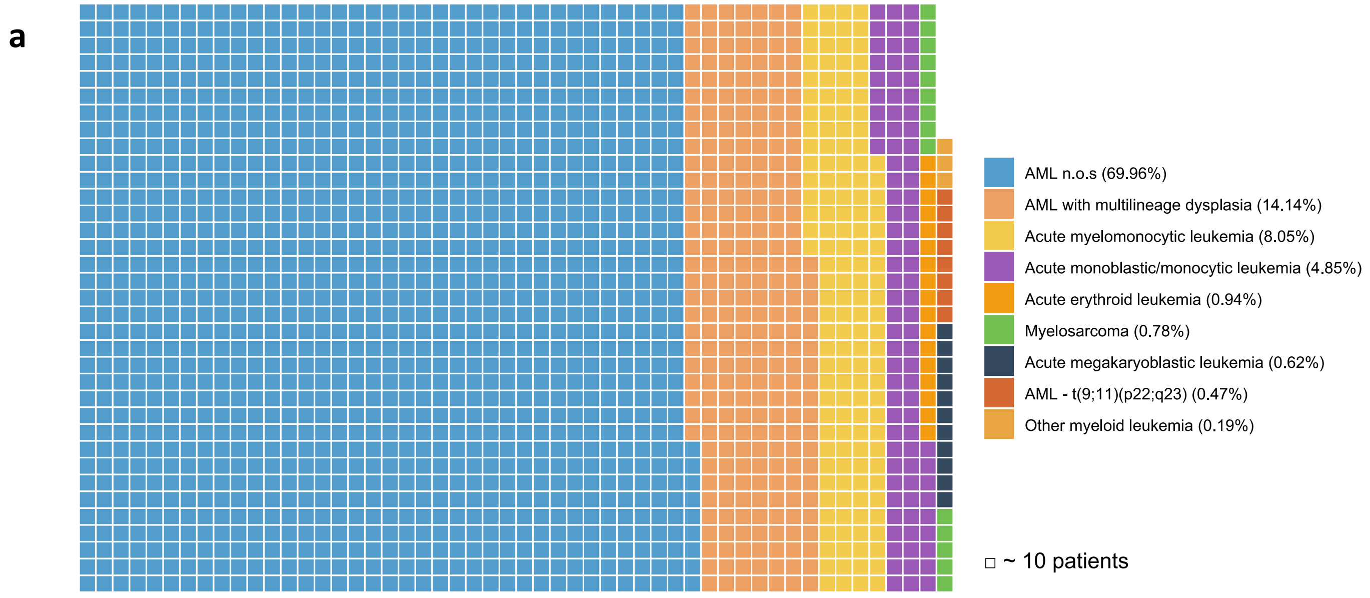

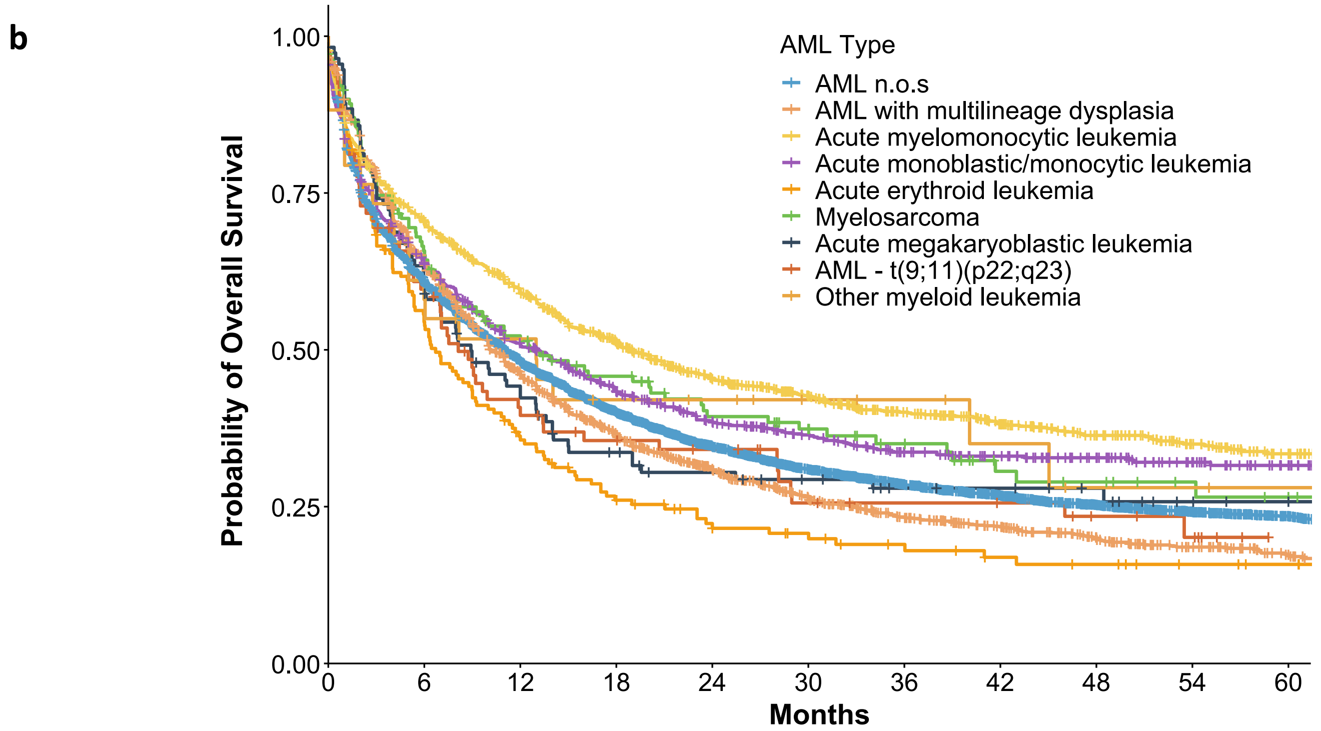

Number at risk

	0	6	12	18	24	30	36	42	48	54	60
AML n.o.s	12697	7599	5734	4247	3196	2471	1923	1488	1100	744	458
AML with multilineage dysplasia	2566	1588	1079	754	553	395	288	222	159	104	63
Acute myelomonocytic leukemia	1462	1005	811	631	488	394	306	241	190	132	81
Acute monoblastic/monocytic leukemia	881	547	414	323	258	209	166	135	105	73	48
Acute erythroid leukemia	171	90	58	40	29	24	19	15	13	9	7
Myelosarcoma	141	89	67	55	42	35	27	18	16	12	10
Acute megakaryoblastic leukemia	113	68	47	33	27	22	17	16	13	7	5
AML - t(9;11)(p22;q23)	85	49	32	26	23	15	13	12	8	6	0
Other myeloid leukemia	34	19	16	12	11	8	7	5	3	3	2

Supplement Figure 5: Distribution (a) and survival (b) of patients by AML-type (n=18 150). Patients with acute myelomonocytic leukaemia had the longest median overall survival at 19·0 months (95% CI: 16·9–21·6), followed by those with acute monoblastic/monocytic leukaemia (13·0 months, 95% CI: 10·5–15·6). In contrast, acute erythroid leukaemia showed the shortest median overall survival (6·7 months, 95% CI: 5·4–9·2). Patients diagnosed with AML not otherwise specified (n.o.s.) had a median survival of 11·0 months (95% CI: 10·7–11·5).

Supplement Figure 6: Overall survival of patients with AML by therapy type and age groups

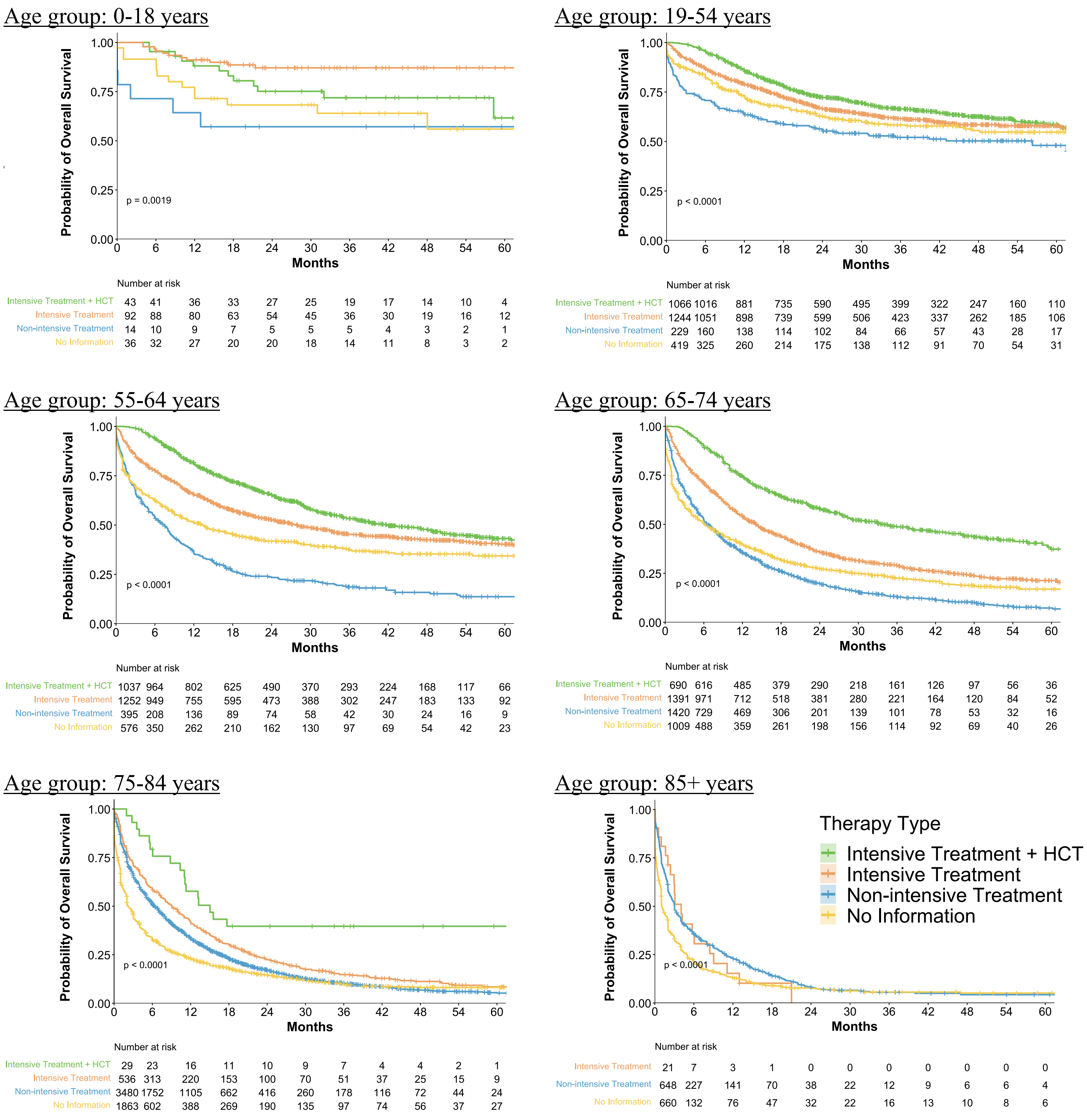

Supplement Figure 6: Overall survival of patients with AML, stratified by treatment and age groups. A p-value of <0·05 (or <0·0125 after multiple testing correction using Bonferroni-method) indicates that survival rates differ between treatment groups. *Abbreviation: HCT: allogeneic hematopoietic stem cell transplantation*

Supplement Figure 7: Treatments in patients with AML ≥75 years

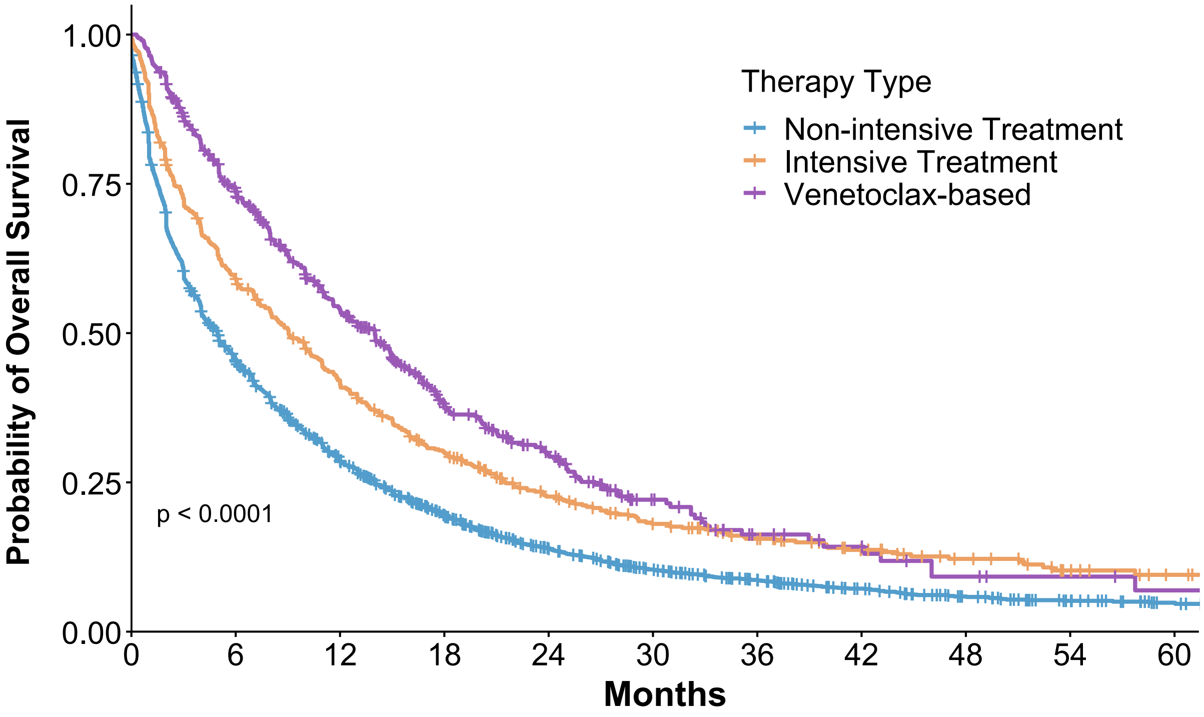

Number at risk

Non-intensive Treatment	3622	1626	1014	607	380	245	170	112	71	45	25
Intensive Treatment	586	343	239	165	110	79	58	41	29	17	10
Venetoclax-based	506	353	232	125	74	37	20	13	7	5	3

Supplement Figure 7: Overall survival of adult patients (≥75 years) with AML treated either with intensive or non-intensive regimens (blue and orange) or Venetoclax-based non-intensive regimens (purple). A p-value of <0·05 (or <0·016 after multiple testing correction using Bonferroni-method) indicates that survival rates differ between treatment groups.

Supplement Figure 8: Overall survival of patients with ALL by age and sex

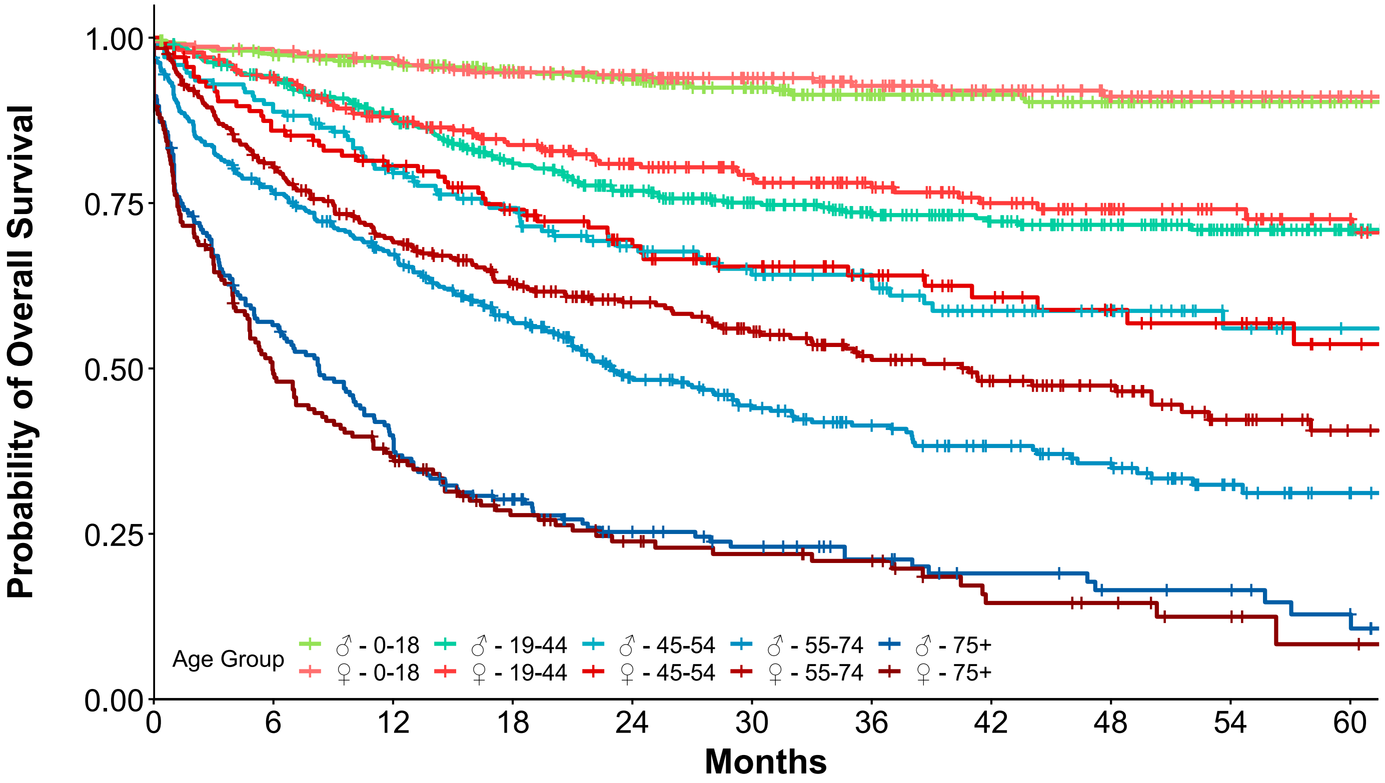

Number at risk

♂ - 0-18	464	442	411	372	323	276	223	182	135	95	62
♀ - 0-18	295	285	276	242	218	184	148	119	99	67	44
♂ - 19-44	488	449	394	326	272	224	180	148	116	81	44
♀ - 19-44	270	245	214	186	161	135	111	88	65	51	38
♂ - 45-54	170	150	127	107	85	72	62	45	35	21	17
♀ - 45-54	136	116	101	86	71	58	45	35	30	25	14
♂ - 55-74	397	299	252	192	135	108	84	65	50	28	16
♀ - 55-74	324	255	210	170	138	117	89	72	57	33	19
♂ - 75+	204	113	79	54	38	30	22	16	12	11	7
♀ - 75+	173	83	59	38	27	23	20	11	9	5	2

Supplement Figure 8: Overall survival of patients with ALL stratified by age group and sex. Blue-green colours represent male patients (n=1 724), while female patients (n=1 198) are represented by red lines. Sex-specific differences reached significance in the age group 55-74 [median OS male patients: 23·0 months (20·5-30·0) vs. median OS female patients: 40·9 months (30·4-52·9), p<0·0001].

Supplement Figure 9: Overall survival of patients with B-ALL by age group and TKI-treatment status

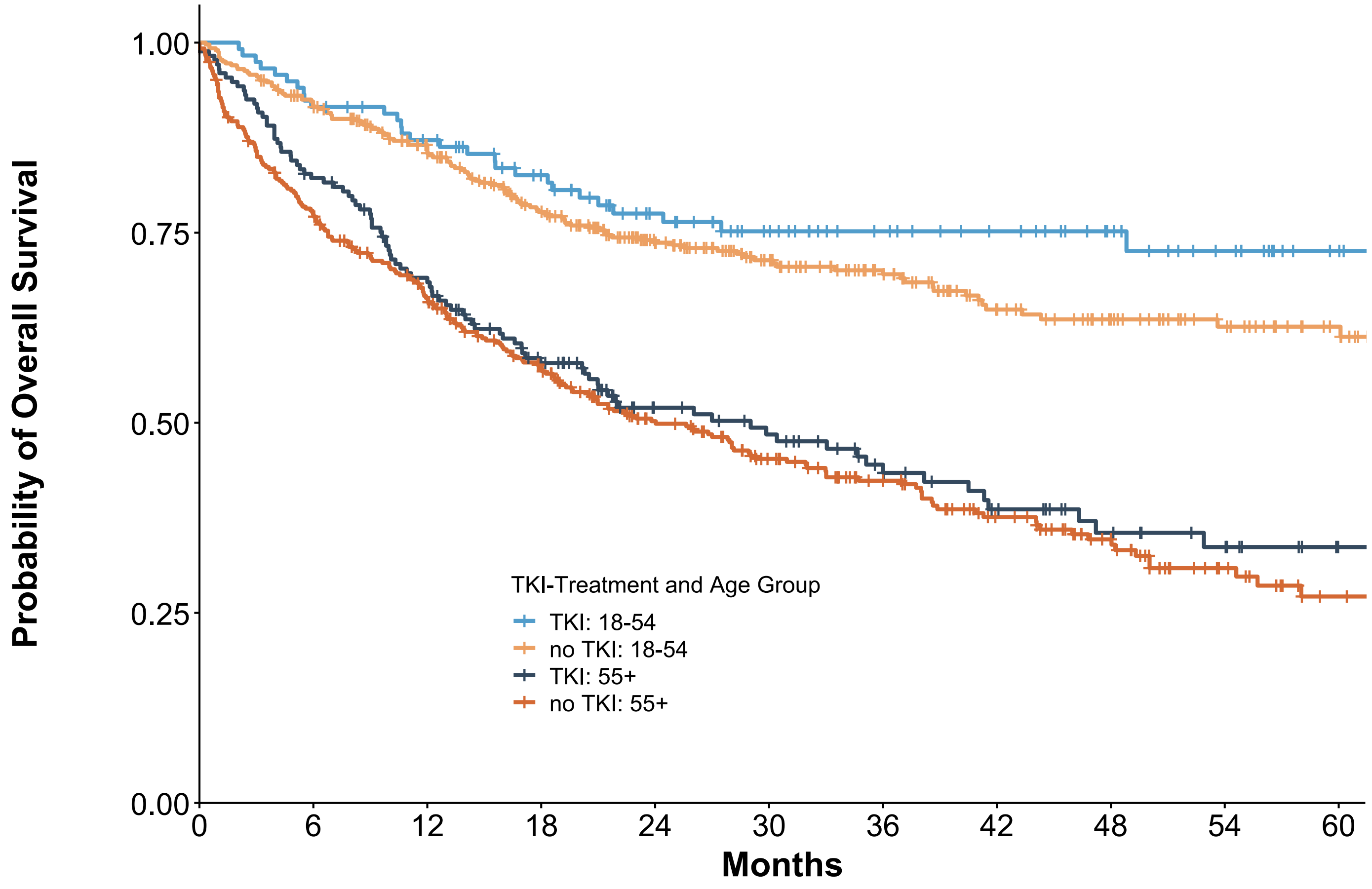

Number at risk

TKI: 18-54	118	108	99	85	69	57	47	39	31	23	13
no TKI: 18-54	402	363	317	263	215	170	135	104	87	66	47
TKI: 55+	174	142	115	88	61	54	41	31	23	18	9
no TKI: 55+	389	295	244	194	148	117	94	71	50	29	15

Supplement Figure 9: Overall survival of patients with B-ALL by age group and TKI-treatment status. Survival in patients treated with TKI and those that not received TKI-treatment in B-ALL was comparable in patients aged 18-54 years (3y-OS 75·2% vs 69·5%, p=0·16) and in those aged 55 years or older (3y-OS 42·2% and 42·5%, p=0·42). TKI treatment is indicated for patients with a Philadelphia chromosome positive ALL. All patients also received additional chemotherapy. *Abbreviation: TKI: tyrosine-kinase inhibitor*

Supplement Figure 10: Overall survival of patients with ALL undergoing allogeneic hematopoietic stem cell transplantation by age group

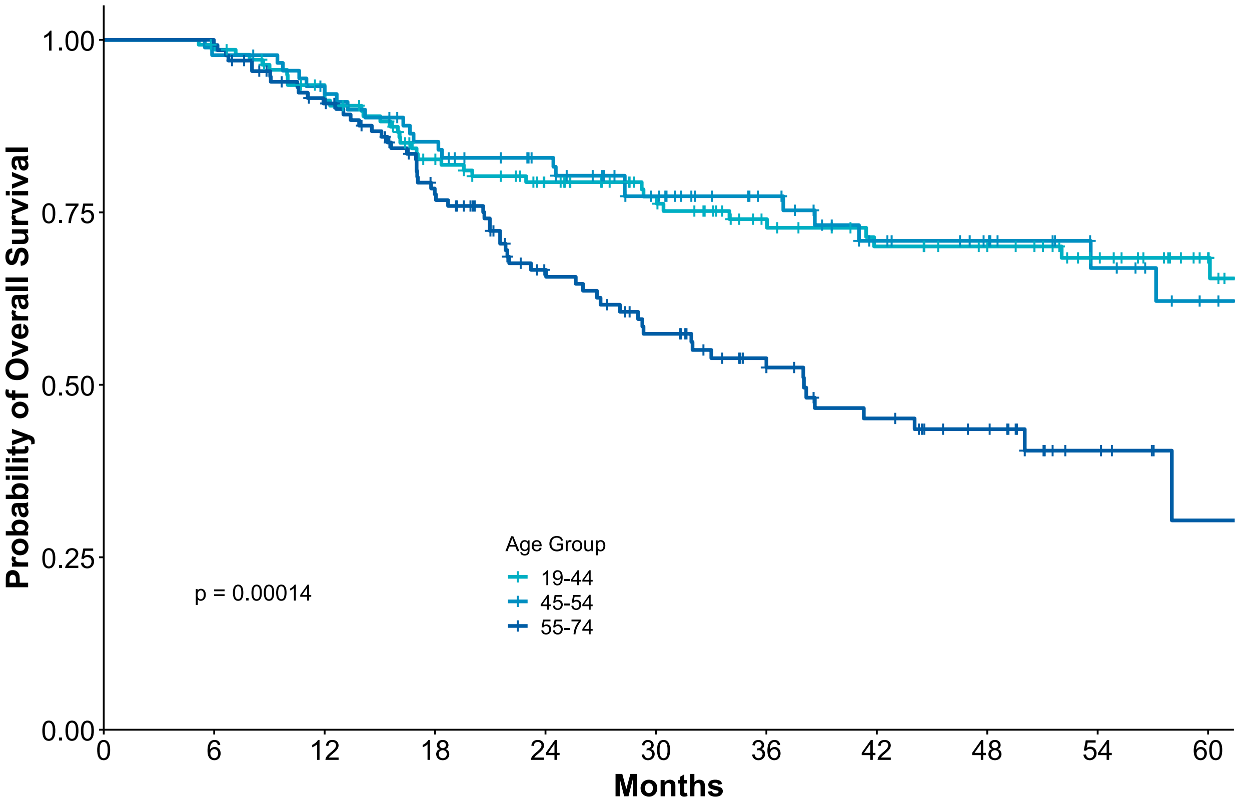

Number at risk

19-44	140	138	125	103	88	74	59	52	48	38	25
45-54	90	88	82	73	64	50	39	29	24	17	11
55-74	133	132	115	92	66	54	40	30	19	8	3

Supplement Figure 10: Overall survival of patients with ALL treated with chemotherapy and allocated to allogeneic hematopoietic stem cell transplantation by age group. Survival is significantly lower in patients aged 55-74 years (3y-OS: 74·0% vs· 77·3% vs· 52·5%, p<0·001). A p-value of <0·05 (or <0·016 after multiple testing correction using Bonferroni-method) indicates that survival rates differ between treatment groups.

Supplement Table 1: Raw and age-standardized incidence rates by standard population

Acute Leukaemia	Standard population	Incidence rate per 100 000
**AML**	None - raw incidence	5·26
**AML**	European standard population 2013	4·72
**AML**	European standard population 1976	3·24
**AML**	US standard population 2000	3·51
**AML**	WHO standard population 2000-2025	2·67
**ALL**	None - Raw Incidence	1·30
**ALL**	European standard population 2013	1·36
**ALL**	European standard population 1976	1·57
**ALL**	US standard population 2000	1·54
**ALL**	WHO standard population 2000-2025	1·70

Supplement Table 1: Raw incidence and mean age-standardized incidence rates (based on 2016-2020) by 4 different standard populations for AML and ALL. *Abbreviations: AML: acute myeloid leukaemia, ALL: acute lymphoblastic leukaemia, US: United States, WHO: World Health Organization*

Supplement Table 2: Uni- and multivariate analysis of survival in AML

Term	*HR (95% CI) Univariate*	*HR (95% CI) Multivariate*	*p-value Univariate*	*p-value Multivariate*
Age at diagnosis	1·05 (1·05–1·05)	1·03 (1·03–1·04)	< 0·0001	< 0·0001
Sex: Female vs Male	0·93 (0·90–0·97)	0·95 (0·92–0·98)	0·00019	0·0046
C92.3 vs. C92.0	0·86 (0·69–1·05)	0·92 (0·75–1·14)	0·14	0·44
C92.5 vs. C92.0	0·73 (0·68–0·79)	0·90 (0·84–0·97)	< 0·0001	0·0035
C92.6 vs. C92.0	1·09 (0·85–1·40)	1·09 (0·84–1·40)	0·50	0·52
C92.7 vs. C92.0	0·87 (0·57–1·34)	0·70 (0·45–1·07)	0·54	0·098
C92.8 vs. C92.0	1·06 (1·00–1·11)	0·93 (0·89–0·98)	0·034	0·0086
C93.0 vs. C92.0	0·88 (0·80–0·95)	1·06 (0·97–1·15)	0·0024	0·21
C94.0 vs. C92.0	1·31 (1·10–1·55)	1·29 (1·09–1·53)	0·0021	0·0030
C94.2 vs. C92.0	1·02 (0·82–1·27)	1·32 (1·06–1·64)	0·87	0·015
Intensive Treatment vs. non-IT.	0·43 (0·41–0·45)	0·72 (0·68–0·75)	< 0·0001	< 0·0001
HCT vs. non-IT	0·26 (0·24–0·28)	0·48 (0·45–0·52)	< 0·0001	< 0·0001
No Information (Treatment) vs. non-IT	0·99 (0·95–1·03)	1·17 (1·12–1·23)	0·61	< 0·0001

Supplement Table 2: Univariate and multivariate Cox models evaluated the impact of age (as a continuous variable), sex, and AML subtypes on overall survival in AML. Older age was consistently linked to higher mortality, while female sex was associated with slightly lower risk of death. We further examined this finding using an interaction model (age and sex). Older age was linked to poorer survival (HR 1.045, 95% CI 1.043–1.047, p <0.0001). Female sex was initially associated with lower mortality (HR 0.749, 95% CI 0.601–0.933, p=0.0092), but the significant age–sex interaction (HR 1.003, 95% CI 1.000–1.006, p=0.034) suggests that the mortality risk increases more rapidly with age in women, gradually diminishing their initial survival advantage. Among AML subtypes C92.0 was used as reference, regarding therapies non-intensive treatments were used as reference.
Abbreviations: HR: Hazard Ratio; CI: Confidence Interval; ICD-10: International Classification of Diseases, 10th Revision; non-IT: non-intensive treatment; C92.3: Myelosarcoma; C92.5: Acute Myelomonocytic Leukaemia; C92.6: AML with t(9;11)(p22;q23); C92.7: Other myeloid leukaemia; C92.8: AML with multilineage dysplasia; C93.0: Acute Monoblastic/Monocytic Leukaemia; C94.0: Acute Erythroid Leukaemia; C94.2: Acute Megakaryoblastic Leukaemia

Supplement Table 3: Uni- and multivariate analysis of survival in ALL

Term	*HR (95% CI) Univariate*	*HR (95% CI) Multivariate*	*p-value Univariate*	*p-value Multivariate*
Age at diagnosis	1·04 (1·04–1·04)	1·04 (1·04–1·04)	<0·0001	<0·0001
Sex: Female vs Male	0·98 (0·86–1·11)	0·85 (0·75–0·97)	0·76	0·015

Supplement Table 3: Univariate and multivariate Cox models evaluated the impact of age (as a continuous variable) and sex on overall survival in ALL. Older age was consistently linked to higher mortality, while female sex was associated with slightly lower risk of death in multivariate, but not univariate analysis. We further explored the effect of age and sex using an interaction model. Higher age was significantly associated with worse survival (HR 1.038, 95% CI 1.033–1.042, p<0.0001). Female sex was linked to lower mortality at baseline (HR 0.589, 95% CI 0.353–0.982, p =0.042). However, the interaction between age and sex was not statistically significant (HR 1.006, 95% CI 0.998–1.014, p=0.14), suggesting that the age-related increase in mortality does not differ meaningfully between sexes in this model. *Abbreviations: HR: Hazard Ratio; CI: Confidence Interval*

Supplement Table 4: Uni- and multivariate analysis of income-related survival in AML

Term	*HR (95% CI) Univariate*	*HR (95% CI) Multivariate*	*p-value Univariate*	*p-value Multivariate*
Age at diagnosis	1·05 (1·05–1·05)	1·05 (1·05–1·05)	< 0·0001	< 0·0001
Sex: Female vs Male	0·92 (0·89–0·95)	0·93 (0·89–0·96)	< 0·0001	< 0·0001
Income: Lower middle vs Low	0·93 (0·89–0·98)	0·99 (0·95–1·04)	0·0056	0·78
Income: Upper middle vs Low	0·96 (0·92–1·01)	1·03 (0·98–1·08)	0·14	0·30
Income: High vs Low	0·91 (0·87–0·96)	0·96 (0·91–1·01)	0·00034	0·11
C92.3 vs. C92.0	0·77 (0·63–0·95)	0·86 (0·70–1·05)	0·012	0·14
C92.5 vs. C92.0	0·75 (0·70–0·81)	0·89 (0·83–0·96)	< 0·0001	0·002
C92.6 vs. C92.0	0·94 (0·73–1·20)	1·17 (0·91–1·50)	0·62	0·21
C92.8 vs. C92.0	1·13 (1·08–1·19)	0·95 (0·90–1·00)	< 0·0001	0·046
C93.0 vs. C92.0	0·87 (0·80–0·95)	1·03 (0·94–1·12)	0·002	0·50
C94.0 vs. C92.0	1·30 (1·11–1·51)	1·31 (1·13–1·53)	0·00090	0·00052
C94.2 vs. C92.0	0·96 (0·77–1·21)	1·35 (1·08–1·70)	0·75	0·0086

Supplement Table 4: Univariate and multivariate Cox models evaluated the impact of age (as a continuous variable), sex, income, and AML subtypes on overall survival based on data from the German Centre for Cancer Registry Data. Among AML subtypes, C92.0 served as the reference category. *Abbreviations: HR: Hazard Ratio; CI: Confidence Interval; ICD-10: International Classification of Diseases, 10th Revision; C92.3: Myelosarcoma; C92.5: Acute Myelomonocytic Leukaemia; C92.6: AML with t(9;11)(p22;q23); C92.8: AML with multilineage dysplasia; C93.0: Acute Monoblastic/Monocytic Leukaemia; C94.0: Acute Erythroid Leukaemia; C94.2: Acute Megakaryoblastic Leukaemia*

Supplement Table 5: Uni- and multivariate analysis of income-related survival in ALL

Term	*HR (95% CI) Univariate*	*HR (95% CI) Multivariate*	*p-value Univariate*	*p-value Multivariate*
Age at diagnosis	1·04 (1·04–1·04)	1·04 (1·04–1·04)	< 0·0001	< 0·0001
Sex: Female vs Male	1·04 (0·94–1·14)	0·91 (0·82–1·00)	0·45	0·045
Income: Lower middle vs Low	0·88 (0·77–1·00)	1·15 (1·01–1·30)	0·043	0·038
Income: Upper middle vs Low	0·82 (0·72–0·94)	1·01 (0·89–1·15)	0·0032	0·90
Income: High vs Low	0·79 (0·69–0·90)	0·96 (0·84–1·09)	0·00055	0·52

Supplement Table 5: Univariate and multivariate Cox proportional hazards models assessing the impact of demographic factors (age, sex) and income groups on overall survival in ALL based on data from the German Centre for Cancer Registry Data. Older age was consistently linked to higher mortality. While sex was not significant in univariate analysis, multivariate results showed a slightly lower risk for females. Higher income was associated with better survival in univariate analysis, but this effect was not significant after adjusting for age and sex. *Abbreviations: HR: Hazard Ratio; CI: Confidence Interval; ICD-10: International Classification of Diseases, 10th Revision*

Supplement Table 6: Uni- and multivariate analysis of GISD-related survival in AML

Term	*HR (95% CI) Univariate*	*HR (95% CI) Multivariate*	*p-value Univariate*	*p-value Multivariate*
Age at diagnosis	1·05 (1·05–1·05)	1·05 (1·05–1·05)	< 0·0001	< 0·0001
Sex: Female vs Male	0·92 (0·89–0·95)	0·93 (0·89–0·96)	< 0·0001	< 0·0001
GISD: Lower middle vs Low	1·10 (1·05–1·16)	1·11 (1·05–1·16)	0·00016	< 0·0001
GISD: Upper middle vs Low	1·09 (1·04–1·15)	1·06 (1·01–1·12)	0·00047	0·017
GISD: High vs Low	1·09 (1·04–1·15)	1·08 (1·03–1·13)	0·00074	0·0030
C92.3 vs. C92.0	0·77 (0·63–0·95)	0·86 (0·70–1·05)	0·012	0·14
C92.5 vs. C92.0	0·75 (0·70–0·81)	0·89 (0·83–0·96)	< 0·0001	0·0016
C92.6 vs. C92.0	0·94 (0·73–1·20)	1·16 (0·90–1·48)	0·62	0·25
C92.8 vs. C92.0	1·13 (1·08–1·19)	0·95 (0·90–1·00)	< 0·0001	0·044
C93.0 vs. C92.0	0·87 (0·80–0·95)	1·03 (0·94–1·12)	0·0023	0·51
C94.0 vs. C92.0	1·30 (1·11–1·51)	1·31 (1·13–1·53)	< 0·00091	< 0·00053
C94.2 vs. C92.0	0·96 (0·77–1·21)	1·34 (1·07–1·68)	0·75	0·010

Supplement Table 4: Univariate and multivariate Cox models evaluated the impact of age (as a continuous variable), sex, GISD, and AML subtypes on overall survival based on data from the German Centre for Cancer Registry Data. For GISD, the least deprived quartile (GISD low) was used as reference. Among AML subtypes, C92.0 served as the reference category. *Abbreviations: HR: Hazard Ratio; CI: Confidence Interval; GISD: German Index for Social Deprivation; ICD-10: International Classification of Diseases, 10th Revision; C92.3: Myelosarcoma; C92.5: Acute Myelomonocytic Leukaemia; C92.6: AML with t(9;11)(p22;q23); C92.8: AML with multilineage dysplasia; C93.0: Acute Monoblastic/Monocytic Leukaemia; C94.0: Acute Erythroid Leukaemia; C94.2: Acute Megakaryoblastic Leukaemia*

Supplement Table 7: Uni- and multivariate analysis of GISD-related survival in ALL

Term	*HR (95% CI) Univariate*	*HR (95% CI) Multivariate*	*p-value Univariate*	*p-value Multivariate*
Age at diagnosis	1·04 (1·04–1·05)	1·04 (1·04–1·05)	< 0·0001	< 0·0001
Sex: Female vs Male	1·04 (0·93–1·16)	0·92 (0·82–1·03)	0·51	0·16
GISD: Lower middle vs Low	1·11 (0·94–1·31)	1·13 (0·95–1·33)	0·23	0·16
GISD: Upper middle vs Low	1·24 (1·06–1·46)	1·14 (0·97–1·35)	0·0088	0·10
GISD: High vs Low	1·34 (1·14–1·57)	1·07 (0·91–1·25)	0·00035	0·42

Supplement Table 7: Univariate and multivariate Cox proportional hazards models assessing the impact of demographic factors (age, sex) and GISD groups on overall survival in ALL based on data from the German Centre for Cancer Registry Data. For GISD, the least deprived quartile (GISD low) was used as reference. *Abbreviations: HR: Hazard Ratio; CI: Confidence Interval; GISD: German Index for Social Deprivation*
